# Finite element analysis of foot-insole biomechanics for insole design: a systematic review

**DOI:** 10.3389/fbioe.2026.1994273

**Published:** 2026-10-28

**Authors:** Xiaolong Li, Miao Gao, Tony Lin-Wei Chen, Bengang Yu, Fan Yang, Zihan Yang, Yaohua Wang

**Affiliations:** 1 Fashion Accessory Art and Engineering College, Beijing Institute of Fashion Technology, Beijing, China; 2 Department of Biomedical Engineering, The Hong Kong Polytechnic University, Kowloon, Hong Kong SAR, China; 3 Beijing Economic-Technological Development Area (Tongzhou), Beijing, China

**Keywords:** biomechanics, finite element analysis, foot orthosis, insole, lower limb

## Abstract

**Objective:**

To synthesise finite element analysis (FEA) evidence on how insole design features influence foot biomechanics, and to identify implications for insole manufacture and the clinical management of foot disorders.

**Methods:**

Web of Science, ScienceDirect, and PubMed were searched. Studies meeting the eligibility criteria were reviewed in full text. A best-evidence synthesis was used to determine the strength of evidence. For each included study, we extracted and compared the research objective, insole parameters, finite element model configuration, loading and boundary conditions, model validation and outcome measures to identify common modelling and experimental practices.

**Results:**

As of December 2025, this review included 45 research articles. The mean methodological quality score was 23.91; 27 studies were classified as high quality and 18 as low quality. Inter-rater reliability was good (ICC[3, 1] = 0.856, 95% confidence interval 0.752–0.918).

**Conclusion:**

Limited evidence indicates that conforming insoles may reduce plantar pressure, although the optimal conformity index remains unclear. Wedge and arch-support insoles can improve foot alignment, but their parameters require careful tuning to avoid excessive soft-tissue loading. Low-modulus materials and plug-and-hole structures in high-load plantar regions may reduce local pressure. Edge gradients or multiple small plugs and holes may limit secondary pressure peaks. Increasing insole thickness and reducing material stiffness can also relieve plantar pressure, but both strategies appear to have an upper threshold. Evidence remains sparse for nonfeatured, metatarsal, heel and comprehensive insoles, and optimal configurations for wedge, arch-support and multihole or multiplug designs have not been established.

**Systematic Review Registration:**

identifier [CRD420251251042].

## Introduction

Sports-related injuries and pathological conditions can cause common foot problems, including heel pain, plantar fasciitis, abnormal pronation or supination, flat feet and diabetic foot ulcers. These conditions can impair physical function and impose substantial financial costs (Bonanno et al., 2017; Cavanagh et al., 2012). Insoles are positioned directly beneath the foot and can be modified in geometry, material stiffness and thickness to provide cushioning and shock absorption. They are also used clinically as non-invasive interventions to relieve pain, improve gait and correct foot deformity (Kim et al., 2010; Zhang et al., 2017). Insole performance is commonly assessed using plantar-pressure measurements and subjective feedback (Cheung and Zhang, 2008). These approaches cannot estimate internal stress and strain in musculoskeletal tissues (Abdallah and Radwan, 2011; Cheung and Zhang, 2005), and their interpretation is limited by the spatial resolution of pressure-sensing systems and interindividual variation in perceived comfort. Prototype development is also iterative, costly and time-consuming (Abdallah and Radwan, 2011; Chen et al., 2015; Nouman et al., 2023; Nouman et al., 2021; Hannah et al., 2016; Peng et al., 2022). FEA can simulate complex foot-insole interactions under defined loading and boundary conditions (Cheung et al., 2009; Guiotto et al., 2014; Song et al., 2022), allowing non-invasive estimation of internal tissue stress-strain responses that are difficult to measure *in vivo*. It therefore provides a quantitative framework for evaluating insole design and function.

Although systematic reviews and meta-analyses have addressed insole design, footwear FEA and lower-limb FEA (Bonanno et al., 2017; Cheung et al., 2009; Song et al., 2022; Shaw et al., 2018), an integrative review of insole design within an FEA framework remains scarce. Inconsistent insole terminology is one barrier to synthesis (Healy et al., 2010). Insoles may be described as customised, pressure-reducing or therapeutic, although individual designs often serve overlapping functions. This ambiguity complicates comparison across studies. A further challenge is developing models that are both computationally feasible and sufficiently representative of real-world conditions. The validity of insole assessment depends on model fidelity, including the definition of geometry, material properties, loading and boundary conditions. These elements are often simplified or underreported, which limits translation of simulation findings. This review therefore aimed to: (1) systematically evaluate FEA studies of insole design; (2) synthesise the biomechanical effects of key design features on foot tissues; and (3) translate these findings into evidence-informed guidance for daily and clinical use. We also critically summarised commonly adopted modelling strategies and experimental configurations to inform future simulation research.

## Methods

### Protocol and registration

This systematic review followed the Preferred Reporting Items for Systematic Reviews and Meta-Analyses (PRISMA) guideline (Ardern et al., 2022). The review protocol was prospectively registered with the International Prospective Register for Systematic Reviews (PROSPERO) under the registration number CRD420251251042.

### Search strategy

Systematic searches of the Web of Science, ScienceDirect, and PubMed databases were conducted using the relevant key phrase (Table 1). The search within Web of Science encompassed its Core Collection, along with specialized indices including Inspec, MEDLINE, BIOSIS Previews, Chinese Science Citation Database, Korean Journal Database, Grants Index, and SciELO Citation Index. Database-specific filters (e.g., publication year and article type) were applied manually to refine the search results. The dates ranged from January 2000 to December 2025, and the results were limited to research articles in Chinese and English. After duplicates were removed, titles and abstracts were screened for relevance, and the accessibility of each study was confirmed. Records were managed in Microsoft Excel 2022 (Microsoft Corporation, Redmond, Washington, USA). Two assessors (Gao and Li) independently conducted full-text screening; disagreements were resolved through discussion with the corresponding author (Yang).

**TABLE 1:** Search strategy.

Databases	Search key phrases and boolean operators
Web of Science (Web of Science Core Collection, Inspec, MEDLINE, BIOSIS Previews, Chinese Science Citation Database, Korean Journal Database, Grants Index, SciELO Citation Index)	1. (“foot orthos*” OR insole* OR “shoe insert*”) 2. (“finite element” OR FEA OR FEM) AND (analys* OR model* OR simulat*) 3. (foot OR “lower limb” OR biomechanic* OR pressure OR stress OR strain) 4. #1 AND #2 AND #3
ScienceDirect	1. (“foot orthoses” OR insole OR “shoe insert”) AND (“finite element analysis” OR FEA) AND (“foot biomechanic” OR pressure OR stress OR strain)
PubMed	1. (“foot orthos*” OR insole* OR “shoe insert*”) AND (“finite element” OR FEA OR FEM) AND (analys* OR model* OR simulat*) AND (foot OR “lower limb” OR biomechanic* OR pressure OR stress OR strain)

The ScienceDirect database imposes constraints on the number of Boolean operators and the use of truncation within a single query line, necessitating the consolidation of the strategy into a single direct search.

### Eligibility criteria

Studies were included if they met all of the following criteria: (1) FEA was the primary study method, rather than a tool used only for validation or development; (2) the insole was the primary research subject; (3) both foot and insole finite element models were included; (4) insole features or parameters were explicitly reported and constituted the primary research focus; and (5) outcomes were biomechanical responses of the foot. Studies focusing only on model refinement, or using the insole solely as a secondary component or validation tool, were excluded.

### Quality assessment

Methodological quality was assessed using the MQSSFE instrument (Wong et al., 2021). This checklist contains 37 items categorized into six domains: (1) study design and presentation of findings (items 1–8); (2) subject recruitment (items 9–12); (3) model reconstruction and configuration (items 13–20); (4) boundary, loading, and simulation conditions (items 21–26); (5) model verification and validation (items 27–31); and (6) model assumptions and validity (items 32–37). Each item was scored as 1 for compliance (‘+') and 0 for non-compliance (‘-'). Before the formal assessment, the two assessors discussed all 37 items with the corresponding author to establish a consistent understanding of the scoring criteria. The two assessors then independently assessed the methodological quality of each included study according to the MQSSFE scoring criteria. Inter-rater reliability for total MQSSFE scores was evaluated with a two-way mixed-effects intraclass correlation coefficient (ICC[3, 1]) (Koo and Li, 2016) and paired-samples t-tests. An ICC >0.75 indicated good reliability, and a p-value >0.05 suggested that there was no significant systematic bias between assessors. After the reliability assessment, discrepant items were discussed, and unresolved disagreements were adjudicated by the corresponding author. The final MQSSFE scores were then determined based on the resolved assessments. Since the original MQSSFE did not specify a scoring threshold for classifying methodological quality, this study adopted the quality classification threshold used in the PEDro scale (Reurink et al., 2012), setting 60% as the criterion for quality classification. Consequently, studies with a total score of ≥22.2 points (out of a maximum of 37 points) were classified as high-quality studies, while those with a total score of <22.2 points were classified as low-quality studies.

### Data extraction

The data extracted from the included studies are summarized in Supplementary Appendix 1 and comprise author information, geometry acquisition, mesh type, simulated movement, material properties, loading conditions, boundary conditions, analysis type, outcome variables, model validation and experimental validation. Data were standardised where possible. Ambiguous or insufficiently reported information was recorded conservatively as ‘Unknown'.

### Insole classification criteria

Insole types were classified according to the primary design feature investigated in each study. The primary design feature was defined as the core design variable explicitly investigated for its effects on biomechanical outcomes. Studies with multiple design features were classified according to the primary feature when it could be clearly identified; otherwise, they were classified as comprehensive insoles. Studies with highly distinctive or innovative features that could not be adequately categorized within the resulting classification scheme were classified as nonfeatured insoles.

### Cross-study comparability assessment

Before evidence synthesis, a cross-study comparability assessment was conducted for studies involving the same insole design category and outcome measure to determine whether differences in modelling and simulation conditions could affect the evidence synthesis. As no standardized method has been established specifically for assessing comparability across lower-limb finite element studies, a comparability assessment table was developed based on relevant previous studies (Hinz et al., 2022; Hammer and Klima, 2019) through discussion among the research team (Supplementary Appendix 2). The assessment table included 4 minor domains (model, material properties, participants, and loading conditions) and 5 major domains (health status, control group, analysis type, model Dimensions, and movement). Accordingly, cross-study comparability was classified into three categories:

1. Comparable: The major domains were consistent, with only minor differences in the minor domains.
2. Comparable with caution: The major domains were consistent, but some differences existed in the minor domains.
3. Not directly comparable: Differences were present in the major domains, or substantial differences existed in the minor domains.

### Best evidence synthesis

Meta-analysis was not feasible because of substantial heterogeneity in insole features and parameters, outcome variables, subject models and methodological quality. We therefore used best-evidence synthesis to grade each extracted outcome according to study quality and consistency (Slavin, 1995) (Supplementary Appendix 3). Evidence was classified into five groups (Reurink et al., 2012):

1. Strong evidence: Generally consistent results reported by multiple high-quality studies (≥75% of studies report consistent results).
2. Moderate evidence: Generally consistent results reported by one high-quality study and one or more lower-quality studies; alternatively, generally consistent results reported by multiple low-quality studies (≥75% of studies reporting consistent results).
3. Limited evidence: The results are reported by only one study (either high or low quality).
4. Conflicting evidence: The results are inconsistent across studies (<75% of studies reported consistent findings).
5. No evidence: No randomized controlled trials or nonrandomised controlled clinical trials addressing the evidence were performed. However, this level is not applicable to the studies included in this review and was therefore not considered.

The above grading criteria were applied when all relevant studies addressing the same outcome were considered cross-study comparable. When non-comparable studies were present, only comparable studies were included in the cross-study evidence synthesis and evidence grading. When all relevant studies were considered non-comparable, the evidence was classified as limited, regardless of the direction of the findings.

### Sensitivity analysis

Sensitivity analyses were conducted at two levels: (1) sensitivity of methodological quality classification to inter-rater scoring differences; and (2) sensitivity of the best-evidence synthesis to alternative methodological quality classifications.

## Results

Figure 1 summarises the search and study-selection process. Initial screening identified 365 records from Web of Science, 123 records from PubMed, and 829 from ScienceDirect (1317 records in total). The manuscript records 750 exclusions through database filters (e.g., publication year and article type) and duplicate screening, 440 after title and abstract screening, 2 because full texts were inaccessible, and 80 after full-text screening. Forty-five studies were ultimately included. Studies were classified according to their primary insole design feature: plug or hole-structured insoles (n = 8) (Cao et al., 2021; Shaulian et al., 2021; Chanda and Unnikrishnan, 2018; Geiger et al., 2023; Actis et al., 2008; Shaulian et al., 2022; Luo et al., 2011; Mala and Bisheh, 2025), variable stiffness insoles (n = 8) (Wang et al., 2023; Hu et al., 2023; Zhu et al., 2021; Li and Yang, 2024; Jafarzadeh et al., 2021; Tang et al., 2019; Zhang et al., 2025; Cracknell et al., 2025), wedge insoles (n = 4) (Liu and Zhang, 2013; Zhou et al., 2017; Du et al., 2024; Guo et al., 2016), arch support insoles (n = 4) (Cheung and Zhang, 2008; Peng et al., 2021; Su et al., 2017; Alsaleh et al., 2025), conforming insoles (n = 8) (Zhang et al., 2017; Cheung and Zhang, 2005; Serrato-Pedrosa et al., 2024a; Serrato-Pedrosa et al., 2024b; Hsu et al., 2008; Sarikhani et al., 2016; Goske et al., 2006; Chen et al., 2003), partial insoles (n = 2) (Chen et al., 2015; Zhang et al., 2018), comprehensive insoles (n = 3) (Peng et al., 2022; Zhang et al., 2020a; Zhang et al., 2020b), and nonfeatured insoles (n = 8) (Nouman et al., 2023; Nouman et al., 2021; Ab Rashid et al., 2022; Yang et al., 2022; Niu et al., 2020; Strayer et al., 2020; Wang et al., 2025; Lin et al., 2025).

**FIGURE 1:**
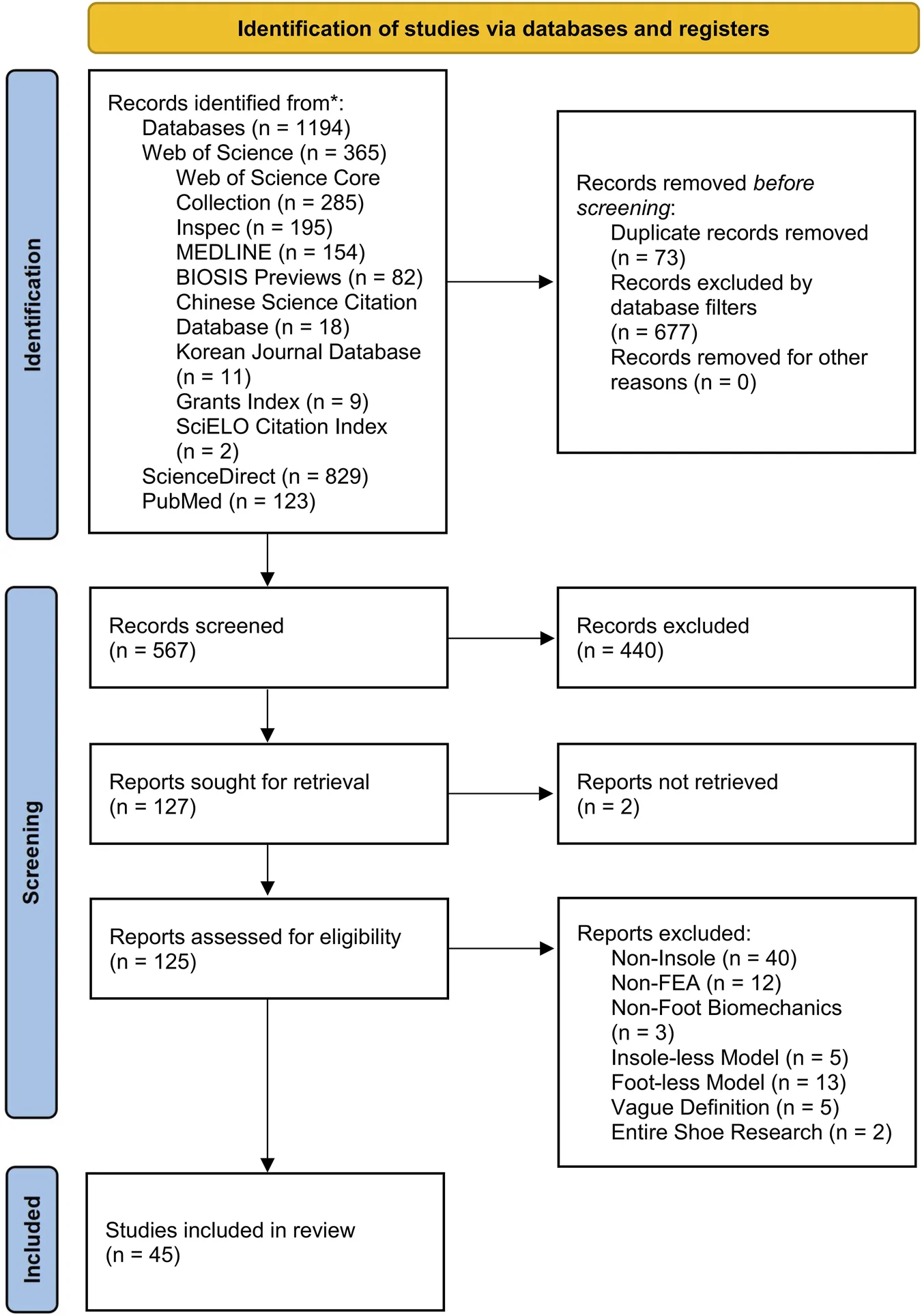
PRISMA literature search process.

**FIGURE 2:**
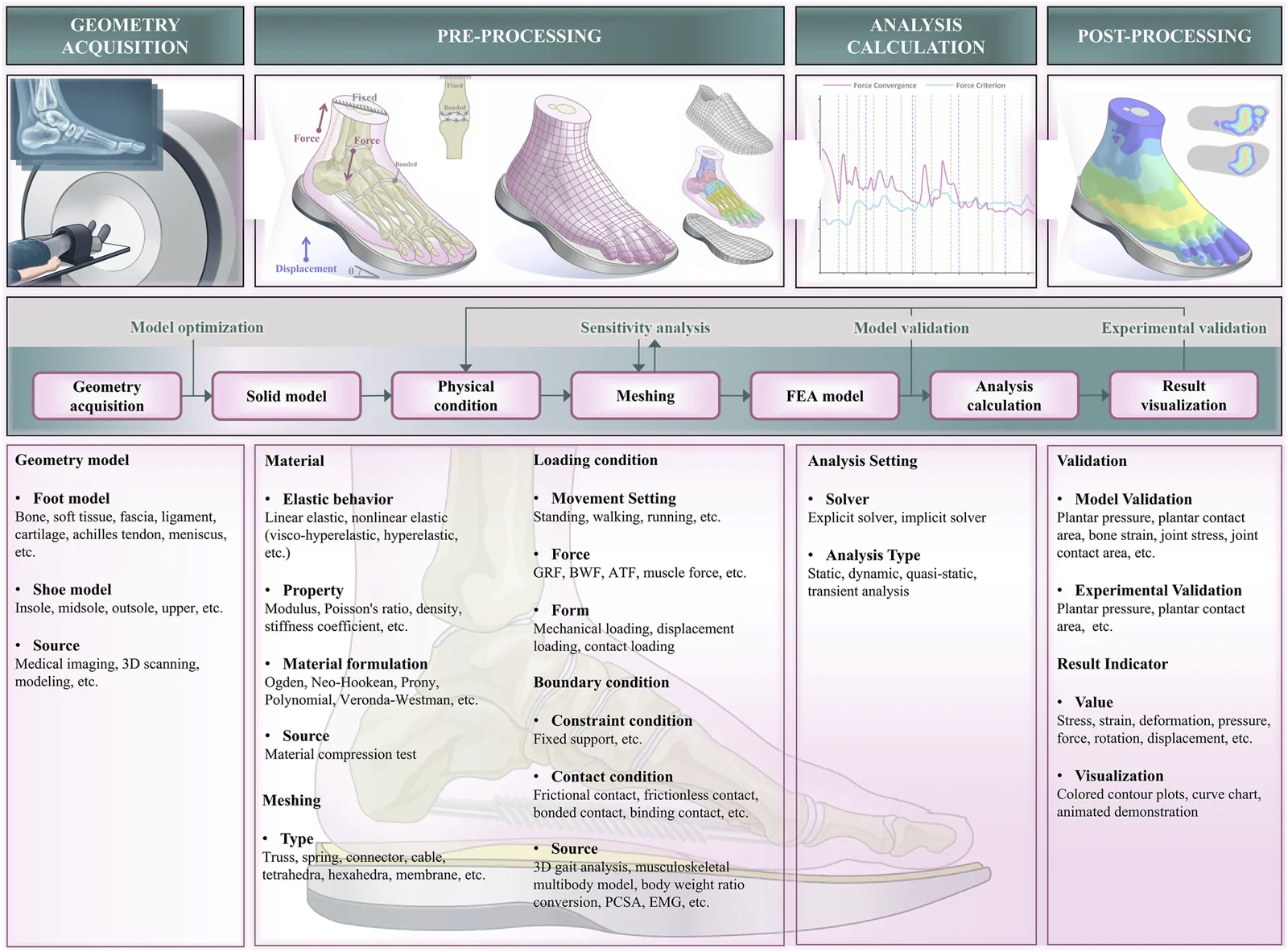
FEA process diagram and experimental condition configuration.

Methodological quality was assessed using the MQSSFE checklist (Supplementary Appendix 4). The mean total score was 23.91 (range 15–35), with 27 studies classified as high quality and 18 as low quality. More than half of the studies adequately reported their research objectives, purpose and planning (items 1–7); used contour plots with consistent colour scales (item 8); reported subject information (items 9 and 10); described geometry acquisition, reconstruction and model settings (items 12–15, 17 and 20); specified material properties (item 19); and described loading and boundary conditions (items 21 and 22). More than half also reported model validation and reliability checks (items 28–30) and discussed implications and translational potential (item 37).

Important deficiencies were also common. Approximately half of the studies omitted demographic characteristics and clinical conditions (item 11), detailed modelling procedures (item 16), or subject-specific loading and boundary data (items 23 and 25). Other frequent limitations were inaccurate loading conditions, including omission of muscle and inertial forces (item 24); failure to report mesh magnitude (item 18), software, analysis type or solver (item 26), mesh sensitivity analysis (item 27), comparisons between predictions and validation data (item 31), and limitations in geometry, material properties or loading conditions (items 32–34). Internal and external validity were also often inadequately discussed (items 35 and 36). Inter-rater analysis identified a constant systematic offset in total MQSSFE scores (paired-samples t-test, p = 0.01), despite good inter-rater agreement (ICC[3,1] = 0.856, 95% CI: 0.752–0.918). Subsequently, the first sensitivity analysis showed that 15 studies had different methodological quality classifications between the individual assessors’ ratings and the final ratings (Supplementary Appendix 5). The second sensitivity analysis showed that the evidence grades remained unchanged across different methodological quality classification schemes for all outcome measures (Supplementary Appendix 6), indicating that the main conclusions of this review were robust.

### FEA process

The FEA workflow can be divided into four stages: geometry acquisition, preprocessing, analysis and postprocessing (Figure 2). Geometry is first obtained by scanning or modelling. Cavity filling, surface smoothing and component coupling are then used to optimise, align and assemble the initial solid model.

During preprocessing, material properties, loading and boundary conditions, and the finite element mesh are defined to approximate the physical scenario. Mesh sensitivity analysis should be used to identify an appropriate mesh density. Model components are commonly simplified within acceptable limits to balance fidelity and computational efficiency.

During analysis, the finite element model and associated engineering files are submitted to a solver for numerical computation. Results are then exported for postprocessing.

Postprocessing includes verification and interpretation of the simulation results. Model validation should precede formal interpretation to establish credibility. Outcomes describing the effect of an insole on lower-limb mechanics include stress, strain, pressure and displacement. Where possible, simulation findings should also be compared with statistical analyses or human testing.

## Finite element insole design

### Nonfeatured insoles

Nonfeatured insoles cannot be categorized and must be discussed independently. Therefore, all outcome variables represent limited evidence. In two studies, insole performance during athletic activities was evaluated. Rashid et al. (Ab Rashid et al., 2022) compared the performance of three badminton insoles. The results indicated that the Li-Ning L6200LA effectively reduced plantar pressure. The insole maximally decreased the peak stress on the metatarsal and calcaneal bones, shifting the stress peak from the fifth metatarsal to the first metatarsal. Due to its high arch support, Li-Ning L6200LA maintained a moderate foot pronation angle, thereby reducing the risk of ligament injury caused by excessive rotation. Yang et al. (Yang et al., 2022) evaluated various components of running shoes, including the insole material, insole thickness, midsole material, and heel cup conforming index. Their results indicated that the insole material had the second-highest contribution to reducing the peak heel pressure. However, the insole thickness had a relatively minor effect on pressure reduction.

Niu et al. (Niu et al., 2020) proposed a layered modular insole in which the thickness in the first metatarsal region was reduced to 1 mm while a thickness of 4 mm was maintained in the arch region. The results reported that this insole design redistributed plantar pressure, alleviating high pressure in the metatarsal region, particularly at the first metatarsal and heel area, thereby reducing ulcer risk. Strayer et al. (Strayer et al., 2020) developed an insole with pressurized bubbles and evaluated the effect of the bubble height (5–10 mm) and bubble stiffness (3–10 MPa) on pressure. The results indicated that increasing the height and stiffness of the bubbles effectively reduced the pressure in the ulcer region, but a higher bubble stiffness also led to increased pressure in the surrounding region, which may result in new ulcers. When the bubble height was 8.3 mm and the stiffness was 5 MPa, there was no contact pressure in the ulcer region, whereas the pressure in the surrounding region was minimized to the greatest extent, thus aiding in the prevention and healing of diabetic foot ulcers. Wang et al. (Wang et al., 2025) investigated the biomechanical effects of an insole with a metatarsal pad, a toe pad, and arch support. They found that the insole reduced plantar fascia stress and redistributed stress in the metatarsals and phalanges to the medial side for a more uniform distribution.

Three studies in this category examined the biomechanical performance of different insole materials. Nouman et al. (Nouman et al., 2021) compared three single-layer insoles (Ethylene Vinyl Acetate (EVA), Amfit, and Thermoplastic Polyurethane (TPU)) and two double-layer insoles (EVA and TPU, Amfit and TPU). They (Nouman et al., 2023) also compared two types of insoles (heel insole and heel-forefoot insole) using materials of varying properties (Plastazote PE, Nora Lunalastike, EVA, and Firm material and a Void design). Both studies reported that softer insole materials offered better pressure-relieving performance, lowering the peak plantar pressure and friction-related stress. The use of such materials could help in the treatment of foot issues related to neuropathic diabetes and decrease callus formation. In their research, the optimal soft materials were EVA (5 MPa, v = 0.4) (Nouman et al., 2021) and Plastazote PE (0.45 MPa, v = 0.38) (Nouman et al., 2023). Although these two studies have highlighted the advantages of compliant materials, Lin et al. (Lin et al., 2025) reported a seemingly contradictory finding. By analysing insoles with varying stiffness levels (100, 260, 1000, 3000, 5000, and 500,000 MPa), they observed that increasing insole stiffness reduced foot displacement while also decreasing the load on the plantar surface and associated bones. However, the authors attributed this discrepancy to simplifications in their model and loading conditions.

### Plug or hole-structured insoles

Five studies evaluated hole-structured insoles (Cao et al., 2021; Shaulian et al., 2021; Chanda and Unnikrishnan, 2018; Luo et al., 2011; Mala and Bisheh, 2025), two studies examined plug-structured insoles (Geiger et al., 2023; Actis et al., 2008), and one study assessed both plug- and hole-structured insoles (Shaulian et al., 2022). Specifically, these structures can be categorized into multihole, multiplug, single-hole, and single-plug structures. Single holes and single plugs typically cover large areas, sometimes spanning the entire metatarsal region, heel region, or larger regions.

Multiple small, compliant plugs or holes can redistribute plantar pressure when positioned beneath high-pressure regions. For multihole structures, Cao et al. (Cao et al., 2021) reported a pressure-reducing configuration with a 5 mm diameter, 6 mm depth and 2 mm interval. For multiplug structures, Actis et al. (Actis et al., 2008) reported a configuration with 4 mm diameter plugs, 1 mm intervals, 70% penetration of insole thickness, 7–9 plugs and Poron material. As each configuration was reported by one study, the evidence is limited.

To alleviate high foot pressure and treat foot ulcers, single-hole and single-plug structures are typically placed over active wound sites to reduce loads (Mala and Bisheh, 2025; Erdemir et al., 2005; Wu et al., 2008). Research has indicated that placing large-area soft-plug structures in high-pressure foot regions effectively reduces the loads on those region (Geiger et al., 2023; Actis et al., 2008; Shaulian et al., 2022). Two studies focused on different aspects. Geiger et al. (Geiger et al., 2023) reported that within a stiffness range of 0.5–1.5 MPa, lower single-plug stiffness resulted in lower peak plantar pressure. Actis et al. (Actis et al., 2008) proposed that box-shaped plugs, which do not fully penetrate the insole thickness, could reduce plantar pressure. However, the evidence for this outcome was limited. Single-hole structures that remove a portion of the insole volume prevent contact between soft tissue and the insole at the recess, thereby isolating the high-pressure region of the foot (Shaulian et al., 2021; Chanda and Unnikrishnan, 2018; Shaulian et al., 2022; Luo et al., 2011; Mala and Bisheh, 2025). Unfortunately, due to differences in study quality, cross-study comparability, and outcome measures, only the reduction in soft-tissue stress was supported by moderate evidence, whereas all other outcomes (total stress concentration exposure of soft tissue, strain and strain energy density of heel pad in high-pressure regions (Shaulian et al., 2021; Chanda and Unnikrishnan, 2018; Luo et al., 2011; Mala and Bisheh, 2025)) were supported by limited evidence. Three studies (Shaulian et al., 2021; Luo et al., 2011; Mala and Bisheh, 2025) reported that single-hole structures that did not fully penetrate the insole thickness, including cylindrical, conical and irregularly shaped structures, could reduce foot loading. However, some of the single-hole insole designs proposed by Shaulian and Luo et al. (Shaulian et al., 2021; Luo et al., 2011) employed a different pressure-reduction mechanism. Specifically, the localized holes in these insoles increased the contact area by conforming to the heel ulcer region, thereby redistributing plantar pressure. Furthermore, while single-hole and single-plug structures benefit the foot, secondary pressure peaks are generated at the deges of such structures, increasing the risk of new ulcer formation in the surrounding region (Erdemir et al., 2005). To address this issue, Shaulian et al. (Shaulian et al., 2022) employed material gradient variations at the edges of plugs and holes, ensuring reduced pressures in local regions while effectively mitigating adverse effects in surrounding regions. Actis et al. (Actis et al., 2008) noted that the use of multiplug structures can reduce these edge effects. Furthermore, Geiger et al. (Geiger et al., 2023) emphasized that the area of a single plug should be slightly larger than that of the high-pressure region to prevent the emergence of secondary pressure peaks.

### Variable stiffness insoles

Four studies (Wang et al., 2023; Hu et al., 2023; Zhu et al., 2021; Zhang et al., 2025) focused exclusively on variable stiffness insoles, whereas Four other studies considered additional parameters, including the full conforming index (Li and Yang, 2024; Tang et al., 2019; Cracknell et al., 2025), variable thicknesses (Li and Yang, 2024), and variable shapes (Jafarzadeh et al., 2021). Moderate evidence (Zhu et al., 2021; Jafarzadeh et al., 2021) indicates that an optimized variable stiffness for insoles significantly reduces the peak plantar pressure. Limited evidence (Wang et al., 2023; Hu et al., 2023; Zhu et al., 2021) suggests that it can also reduce stress in the soft tissue, bones and cartilage. Further consideration of the conformity index, variable thickness, and variable shape may enhance the pressure-reducing effects of insoles (Li and Yang, 2024; Jafarzadeh et al., 2021; Tang et al., 2019; Cracknell et al., 2025). Although the available evidence is limited due to heterogeneity across studies, the findings suggest that combining variable stiffness with shape-based design may have potential for further improving the pressure-reducing performance of insoles. For variable stiffness insoles, the material properties in different regions are adjusted on the basis of plantar pressure data. In particular, low-modulus materials are used in high-pressure regions to optimize the pressure distribution at the foot-insole interface. To derive spatial distributions of elastic modulus, five studies (Zhu et al., 2021; Li and Yang, 2024; Jafarzadeh et al., 2021; Tang et al., 2019; Cracknell et al., 2025) employed iterative optimization algorithms designed to minimize peak plantar pressure. In contrast, three studies (Wang et al., 2023; Hu et al., 2023; Zhang et al., 2025) predefined multiple variable-stiffness schemes, in which effective modulus values were achieved by adjusting the wall thickness of porous units. The performance of these schemes was compared to identify the optimal insole design. Overall, low-modulus materials were deemed optimal for the metatarsal and heel regions, with values concentrated near 0–4.7 MPa.

One study (Tang et al., 2019) assigned near-zero modulus values to regions of high plantar loading. Such modulus values may be difficult to achieve in practical manufacturing. This finding also highlights a limitation of numerically optimized insole design, namely, that optimization algorithms may generate material parameters beyond the feasible range of real-world materials. Future studies should first characterize the feasible performance ranges and mechanical limits of candidate materials through experimental testing and incorporate these material constraints into the optimization algorithms. This approach could improve the manufacturability of optimized designs, prevent material failure during practical use due to exceeding their load-bearing capacity, and facilitate the translation of computationally optimized designs into practical and clinical applications.

### Wedge insoles

Three studies (Liu and Zhang, 2013; Zhou et al., 2017; Du et al., 2024) evaluated lateral wedge insoles and one study (Guo et al., 2016) evaluated medial wedge insoles. The results indicate that lateral wedge insoles can reduce stress on the medial femoral cartilage and medial meniscus, as well as the total contact force, thereby redistributing internal knee joint loads (Liu and Zhang, 2013), which may aid in recovery from osteoarthritis and reduce plantar pressure and cuneonavicular joint stress (Du et al., 2024). However, although lateral wedge insoles show benefits, their use is often associated with increased loads on other tissues. For instance, the total contact force on the lateral meniscus increases (Liu and Zhang, 2013). The contact pressure and stress in the subtalar joint increase (Zhou et al., 2017; Du et al., 2024), resulting in foot and ankle pain. The peak metatarsal stress shifts from the second metatarsal to the fourth and fifth metatarsals, resulting in new fracture risks. The stress on key arch-maintaining ligaments and the plantar fascia generally increases, increasing the risk of rupture (Du et al., 2024). Medial wedge insoles may redistribute plantar pressure toward the heel, first metatarsal, and lateral longitudinal arch regions, while reducing stress in the medial ankle ligaments and plantar fascia, as well as in the calcaneus, talus, and first to third metatarsals (Guo et al., 2016). Given the different outcomes assessed and the limited number of studies for each outcome, the evidence was limited. Furthermore, relevant studies suggest that the wedge angle should remain within a moderate range, as excessively steep wedges can cause foot discomfort (Park et al., 2017; Kerrigan et al., 2002). Liu and Du et al. reported that a 5° lateral wedge angle produced favorable responses in knee joint loading and plantar pressure (Liu and Zhang, 2013; Du et al., 2024). Zhou et al. reported that an 8° lateral wedge angle could avoid excessive load concentration (Zhou et al., 2017). Guo et al. found that a medial wedge angle of 7.5°–8° resulted in relatively low levels of plantar pressure and stress in the foot bones, ligaments, and plantar fascia (Guo et al., 2016).

Although different studies reported effective wedge angles, the lack of a basis for direct cross-study comparison means that an optimal angle range for lateral or medial wedge insoles cannot currently be determined. In addition, the wedge angles used across studies of lateral wedge insoles varied considerably; therefore, it remains unclear how foot loading changes with gradual changes in wedge angle. Future studies should expand the coverage of subject-specific models and systematically evaluate different outcome measures while minimizing methodological heterogeneity across studies. Further research should then evaluate the effects of different medial and lateral wedge angles on the biomechanical responses of feet with pronation, supination, and knee osteoarthritis, thereby providing a more reliable basis for determining appropriate wedge-angle ranges for different populations and clinical contexts.

### Arch support insoles

One study (Peng et al., 2021) exclusively evaluated arch support insoles, whereas three studies (Cheung and Zhang, 2008; Su et al., 2017; Alsaleh et al., 2025) also considered insole stiffness (Cheung and Zhang, 2008; Su et al., 2017), material (Alsaleh et al., 2025), thickness (Cheung and Zhang, 2008; Alsaleh et al., 2025), heel cup height (Alsaleh et al., 2025), as well as sole stiffness and sole thickness (Cheung and Zhang, 2008). The results indicate that increasing the arch support height of insoles is beneficial for correcting flatfoot deformities and can also partially alleviate high foot pressure (Cheung and Zhang, 2008; Peng et al., 2021; Su et al., 2017; Alsaleh et al., 2025) and fascia loads (Peng et al., 2021). However, excessively large arch support height may cause foot discomfort (Peng et al., 2021; Su et al., 2017; Alsaleh et al., 2025). Notably, for a given arch support height, greater insole stiffness is correlated with better orthotic performance, whereas softer insoles provide superior pressure relief. Thus, the stiffness of arch support insoles must be carefully determined to ensure effective orthotic function without inducing unnecessary loads. Additionally, Two studies (Cheung and Zhang, 2008; Alsaleh et al., 2025) reported that arch support height contributes most significantly to reducing peak plantar pressure, followed by insole stiffness (material), whereas insole thickness has a weaker effect.

Potential challenges associated with the use of arch support insoles have also been reported. Limited evidence suggests that a high arch support (33 mm) increases the peak stress on major foot ligaments and cartilage in the midfoot and ankle joints (Su et al., 2017). Similarly, a high arch support (48 mm) may impose a load on the central portion of the plantar fascia (Peng et al., 2021). Finally, a high arch support (42 mm) increases peak stress and strain in the foot (Alsaleh et al., 2025). In summary, although the exact impact of the arch support height on the foot cannot be quantified, an excessively tall arch support height is clearly undesirable.

An optimal arch support height range has not yet been established because of inconsistencies in insole support heights or types and foot characteristics. Specifically, in three studies, flatfoot models and different arch support heights (42, 45, and 48 mm (Peng et al., 2021); 27, 30, and 33 mm (Su et al., 2017); 30, 37, and 42 mm (Alsaleh et al., 2025) were used. In another study, arch support types were not defined by height but by the weight-bearing condition (full-, half-, and non-weight-bearing (Cheung and Zhang, 2008)). Consequently, future research should investigate a broader range of arch support parameters and specifically evaluate corrective effects for individuals with flat feet or high arches rather than for individuals with normal feet.

### Conforming insoles

Three studies (Serrato-Pedrosa et al., 2024a; Serrato-Pedrosa et al., 2024b; Hsu et al., 2008) evaluated conforming insoles, one study (Chen et al., 2003) assessed conforming insoles with layered materials, and four studies considered additional parameters, including insole thickness (Zhang et al., 2017; Cheung and Zhang, 2005; Sarikhani et al., 2016; Goske et al., 2006), insole stiffness (Zhang et al., 2017; Cheung and Zhang, 2005; Sarikhani et al., 2016; Goske et al., 2006), and heel height (Sarikhani et al., 2016). All the studies consistently reported that conforming insoles effectively reduce foot loading. Limited evidence suggests that conforming insoles can also alleviate high pressure in the metatarsal and plantar fascia regions. However, the optimal conforming index for maximizing plantar pressure reduction remains unclear. In some studies, full-conforming insoles were associated with the greatest reduction in pressure (Cheung and Zhang, 2005; Serrato-Pedrosa et al., 2024a; Serrato-Pedrosa et al., 2024b; Sarikhani et al., 2016; Goske et al., 2006; Chen et al., 2003). In contrast, Zhang et al. (Zhang et al., 2017) reported that half-conforming insoles were most effective for reducing pressure, possibly because they observed abnormal pressure peaks in the heel region when full-conforming insoles were used, although the specific cause remained unclear. Additionally, Hsu et al. (Hsu et al., 2008) suggested a 54% conforming index as the optimal parameter. However, in this study, the conforming index was adjusted only in the heel region, and the entire foot was not considered. Furthermore, in some studies, only flat and full-conforming insoles were compared and the effects of varying the conforming index were not specifically addressed, which may contribute to the inconsistencies in the optimal conforming index. Additionally, multiparameter designs may have influenced the results.

In multiparameter studies of conforming insoles, the results clearly indicate that full-conforming insoles combined with either increased thickness or softer materials effectively alleviate high foot pressure (Zhang et al., 2017; Cheung and Zhang, 2005; Goske et al., 2006). Limited evidence suggests that this combination also reduces bone stress in the forefoot and midfoot regions, as well as fascial strain (Cheung and Zhang, 2005; Goske et al., 2006). Furthermore, some studies have reported that the conforming index is the most important parameter influencing plantar pressure, followed by the insole material (Cheung and Zhang, 2005; Goske et al., 2006).

In summary, although the evidence obtained in this review was limited, existing studies generally support the potential of conforming insoles to reduce foot loading (Chen et al., 2003). When conforming insoles were combined with other pressure-reducing design features, such as variable stiffness (Li and Yang, 2024; Tang et al., 2019), plug (Shaulian et al., 2022), or hole structures (Shaulian et al., 2022; Luo et al., 2011), some studies also observed reduced plantar pressure.

### Comprehensive insoles

Comprehensive insoles incorporate at least two key design features. As a result, they cannot be classified into one of the above single-feature categories. All outcome variables are supported by limited evidence. Zhang et al. (Zhang et al., 2020b) evaluated the arch support height (20, 25, and 30 mm), lateral wedge angle (3, 5, and 7°), and insole stiffness (1.0, 1.5 and 2.5 MPa). The results of this study indicate that arch support contributes most significantly to reducing plantar stress, followed by wedge angle and insole stiffness. Furthermore, the optimal configuration was identified as an insole with a 30 mm arch height, 5° wedge angle, and a stiffness of 1 MPa. This configuration significantly reduced the peak surface and internal stresses in the heel and metatarsal regions, promoted a more uniform plantar stress distribution, and reduced the stress on the navicular-cuneiform joint cartilage and tensile stress in the plantar fascia. Another study (Zhang et al., 2020a) indicated that insole stiffness had the greatest effect on joint cartilage stress, followed by arch support height and wedge angle. However, the optimal configurations for alleviating stress on specific bones were not consistent. Specifically, the aforementioned optimal insole minimizes the cartilage stress associated with arthritis development. In contrast, the configuration minimizing navicular stress was identified as an insole with a 30 mm arch height, a 3° wedge angle, and a stiffness of 2.5 MPa, whereas the configuration that minimized stress on the medial cuneiform bone was identified as an insole with a 20 mm arch height, a 5° wedge angle, and a stiffness of 1.5 MPa. Peng et al. (Peng et al., 2022) evaluated the arch support height (42, 45, and 48 mm), medial wedge angle (0, 2, and 4°), heel cup height (14, 16, and 18 mm), and insole stiffness (3, 5, and 7 MPa). The results of this study indicated that arch support and the medial wedge angle were the primary factors influencing the reduction in the peak plantar pressure, whereas arch support and insole stiffness were the most influential factors for reducing the peak strain in the proximal plantar fascia.

### Partial insoles

Only two studies investigated partial insoles (heel insoles and metatarsal insoles) and provided limited evidence in relevant fields. Chen et al. (Chen et al., 2015) investigated different placements of metatarsal insoles (none, distal, proximal, and most proximal positions, defined by the longitudinal distance from the second metatarsal head) and various insole thicknesses (2.5, 5.1, 7.6, 10.2, and 12.7 mm). The results demonstrated that increasing the insole thickness reduced the peak metatarsal pressure and soft tissue strain, with a thickness of 12.7 mm being the most effective for pressure reduction. With respect to placement, positioning the insole proximally, where its dome apex was 19.5 mm proximal to the metatarsal centre, resulted in a more uniform pressure distribution, significantly lowering both the peak plantar pressure and the peak soft tissue compression strain. However, distal positioning produced the opposite effect. Notably, to achieve the optimal pressure reduction in the metatarsal region, a uniform soft tissue compression pattern under the metatarsal shaft was needed. Zhang et al. (Zhang et al., 2018) evaluated various heel insole configurations, including various insole shapes (circular, horizontal elliptical, and vertical elliptical), thicknesses (2, 3, and 4 mm), and groove shapes (round hole, honeycomb, and groove). Compared with barefoot conditions and test insoles, the optimal insole configuration-a vertical elliptical shape, a honeycomb groove, and a thickness of 4 mm-provided the most effective pressure reduction and alleviated the plantar fascia load.

Overall, insole geometry, including arch support, conformity and wedge angle, together with material stiffness, are crucial parameters for alleviating foot loads (Cheung and Zhang, 2008; Cheung and Zhang, 2005; Peng et al., 2022; Alsaleh et al., 2025; Goske et al., 2006; Zhang et al., 2020b), although their relative contributions warrant further investigation. Additionally, both softer materials and thicker materials can enhance the pressure-reducing performance (Cheung and Zhang, 2008; Cheung and Zhang, 2005; Nouman et al., 2023; Nouman et al., 2021; Peng et al., 2022; Luo et al., 2011; Mala and Bisheh, 2025; Su et al., 2017; Alsaleh et al., 2025; Sarikhani et al., 2016; Goske et al., 2006; Zhang et al., 2020a; Zhang et al., 2020b; Yang et al., 2022). However, these benefits are constrained by optimal ranges. Excessively compliant insoles may bottom out because of inadequate support (Goske et al., 2006). Excessive thickness may not further reduce plantar pressure and can reduce shoe-cavity volume (Chen et al., 2003; Niu et al., 2020), potentially causing pain. Future work should quantify the beneficial and adverse effects of these parameters to define clinically relevant ranges.

## Discussion

### Geometric acquisition and model construction

Imaging modalities for acquiring the model geometries varied across studies. Computed tomography (CT) delineates bony contours clearly at relatively low cost (Sun and Wen, 2010), but provides limited soft-tissue detail and involves ionising radiation (Song et al., 2022). Magnetic resonance imaging (MRI) captures the contours of tendons, ligaments, muscles and other soft tissues (Du et al., 2020; Morales-Orcajo et al., 2016), but image segmentation is expensive and labour-intensive. Three-dimensional surface scanning is an accessible alternative for acquiring surface topology of the foot and shoe under functional conditions, although it cannot characterise internal structures (Sun and Wen, 2010).

Realistic simulations are needed to evaluate insole effects accurately (Cheung et al., 2009). However, such simulations can be time-consuming and computationally costly. Many studies therefore simplify foot structures to improve efficiency. In the reviewed studies, bones, fascia, ligaments, cartilage and bulk soft tissue were often modelled explicitly, whereas tendons, muscles and skin were more frequently represented using non-geometric approaches. Some studies (Shaulian et al., 2021; Shaulian et al., 2022; Luo et al., 2011; Zhang et al., 2025; Goske et al., 2006; Strayer et al., 2020) restricted the model to an anatomical region, such as the rearfoot. Three-dimensional models now predominate over two-dimensional models. Pathological conditions, including diabetic feet with ulcers (Chanda and Unnikrishnan, 2018; Mala and Bisheh, 2025), amputated feet (Guo et al., 2016), flat feet (Peng et al., 2022; Peng et al., 2021; Su et al., 2017) and complex multimorbid deformities (Alsaleh et al., 2025), have also been modelled.

Specifically on the level of modelling granularity across anatomic components, bony structures are normally represented in substantial detail. Most foot models incorporated major foot bones, including the 14 phalanges, 5 metatarsals, and 7 tarsals, while more sophisticated lower limb model comprises the tibia, fibula, patella, and femur. Some studies adopted simplification strategies, such as merging multiple bones into a single rigid body (Nouman et al., 2023; Nouman et al., 2021; Geiger et al., 2023; Tang et al., 2019) or partially merging metatarsals (Yang et al., 2022) and phalanges (Jafarzadeh et al., 2021; Wang et al., 2025), to increase computational efficiency. The representation of ligamentous structures varies considerably across studies, with no consensus on the number or selection of ligaments to be included. However, the plantar fascia is widely considered as a key structure for maintaining arch stability; therefore, it is typically included in foot models. Cartilage is primarily modelled via two approaches. In some studies, cartilage is simplified as a thin solid structure. Larger cartilages, such as knee joint cartilage, can be modelled with greater geometric and material details (Liu and Zhang, 2013; Zhou et al., 2017). Alternatively, a solid cartilage structure is not established. Instead, its lubricating properties are simulated by setting frictionless contact between bones or joints. The bulk foot soft tissue, which merges the muscle bellys, tendons, and other tissues, is typically obtained by subtracting independently modelled structures from the overall model.

Insole models are commonly constructed from the plantar contour (Cheung and Zhang, 2008; Cheung and Zhang, 2005; Nouman et al., 2021; Peng et al., 2022; Geiger et al., 2023; Luo et al., 2011; Tang et al., 2019; Cracknell et al., 2025; Peng et al., 2021; Su et al., 2017; Alsaleh et al., 2025; Serrato-Pedrosa et al., 2024a; Serrato-Pedrosa et al., 2024b; Chen et al., 2003; Zhang et al., 2018; Zhang et al., 2020a; Zhang et al., 2020b). Their contours may be simplified, for example, as rectangular prisms (Chen et al., 2015; Zhou et al., 2017). Furthermore, even in insole-focused studies, the internal space of the entire shoe formed by the insole, upper region, and sole is often neglected. Only two studies included an upper model (Goske et al., 2006; Yang et al., 2022). Most studies did not construct a complete sole and placed the insole directly on the ground (Zhang et al., 2017; Cheung and Zhang, 2008; Nouman et al., 2023; Nouman et al., 2021; Cao et al., 2021; Luo et al., 2011; Mala and Bisheh, 2025; Wang et al., 2023; Hu et al., 2023; Zhu et al., 2021; Li and Yang, 2024; Jafarzadeh et al., 2021; Tang et al., 2019; Zhang et al., 2025; Cracknell et al., 2025; Liu and Zhang, 2013; Du et al., 2024; Guo et al., 2016; Alsaleh et al., 2025; Serrato-Pedrosa et al., 2024a; Serrato-Pedrosa et al., 2024b; Hsu et al., 2008; Chen et al., 2003; Zhang et al., 2018; Zhang et al., 2020a; Zhang et al., 2020b; Niu et al., 2020; Wang et al., 2025; Lin et al., 2025).

### Mesh type and magnitude

As shown in Table 2, bones, cartilage, soft tissues, Achilles tendons, and menisci are typically meshed with tetrahedral elements, whereas the insoles, outsoles, and ground supports can be meshed by both tetrahedral and hexahedral elements. Research indicates that tetrahedral elements possess superior geometric adaptability, making them suitable for complex models (Chen et al., 2010; Gefen et al., 2000; Cheung et al., 2005; Tadepalli et al., 2011; Burkhart et al., 2013; Phan et al., 2021). Hexahedral elements provide better convergence speed and solution accuracy but are difficult to generate, making them suitable only for regular shapes. When the number of nodes in a tetrahedral element increases from four to ten, the solution accuracy approaches that of models with hexahedral elements (Tadepalli et al., 2011). To more accurately simulate stress distributions in the foot and insole, some studies (Geiger et al., 2023; Tang et al., 2019; Zhou et al., 2017; Yang et al., 2022; Strayer et al., 2020) employ these higher-order tetrahedral elements. Ligaments and fascia are typically simplified and modelled as linear elements at corresponding attachment points on the basis of anatomical principles (Zhang et al., 2017; Cheung and Zhang, 2008; Cheung and Zhang, 2005; Chen et al., 2015; Nouman et al., 2023; Peng et al., 2022; Wang et al., 2023; Hu et al., 2023; Li and Yang, 2024; Jafarzadeh et al., 2021; Liu and Zhang, 2013; Zhou et al., 2017; Du et al., 2024; Guo et al., 2016; Peng et al., 2021; Su et al., 2017; Hsu et al., 2008; Chen et al., 2003; Zhang et al., 2018; Zhang et al., 2020a; Zhang et al., 2020b; Ab Rashid et al., 2022; Yang et al., 2022; Niu et al., 2020). Specifically, truss elements are the most commonly used elements to represent ligamentous constraints, followed by the spring, link, and cable elements. Some studies (Zhang et al., 2017; Cheung and Zhang, 2008; Chen et al., 2015; Guo et al., 2016; Su et al., 2017; Ab Rashid et al., 2022) employ multiple linear elements in modeling ligaments to account for the ligament thickness. To further investigate the plantar fascia, five studies (Chen et al., 2015; Peng et al., 2022; Hu et al., 2023; Peng et al., 2021; Su et al., 2017) constructed its three-dimensional morphology using tetrahedral elements. Regarding other tissues, muscles are generally modelled with connector elements (Cheung and Zhang, 2008; Liu and Zhang, 2013; Peng et al., 2021; Zhang et al., 2020a), Achilles tendons with tetrahedral elements (Shaulian et al., 2021; Shaulian et al., 2022), and skin with tetrahedral (Shaulian et al., 2021; Shaulian et al., 2022; Su et al., 2017; Hsu et al., 2008; Ab Rashid et al., 2022) or membrane elements (Peng et al., 2022; Peng et al., 2021).

**TABLE 2:** Statistics of mesh types of finite element model components.

Model components	Mesh types
Foot	4-node Tet, (Nouman et al., 2021) 10-node Tet, (Geiger et al., 2023) Tet (Nouman et al., 2023; Alsaleh et al., 2025), Quadratic element (Mala and Bisheh, 2025)
Heel pad	4-node Quad, (Luo et al., 2011) Quad (Goske et al., 2006), 4-Node Tet (Zhang et al., 2025)
Bone	4-node Tet, (Zhang et al., 2017; Chen et al., 2015; Peng et al., 2022; Hu et al., 2023; Li and Yang, 2024; Cracknell et al., 2025; Du et al., 2024; Guo et al., 2016; Peng et al., 2021; Hsu et al., 2008; Chen et al., 2003; Zhang et al., 2020a; Zhang et al., 2020b; Ab Rashid et al., 2022; Wang et al., 2025; Lin et al., 2025) 10-node Tet, (Tang et al., 2019; Zhou et al., 2017; Yang et al., 2022; Strayer et al., 2020) Tet (Cheung and Zhang, 2008; Cheung and Zhang, 2005; Shaulian et al., 2021; Shaulian et al., 2022; Jafarzadeh et al., 2021; Liu and Zhang, 2013; Su et al., 2017; Sarikhani et al., 2016; Niu et al., 2020)
Cartilage	4-node Tet, (Chen et al., 2015; Hu et al., 2023; Guo et al., 2016; Hsu et al., 2008; Chen et al., 2003; Zhang et al., 2020a; Lin et al., 2025) 10-node Tet,^42 58^ Tet (Cheung and Zhang, 2008; Cheung and Zhang, 2005; Shaulian et al., 2021; Shaulian et al., 2022; Liu and Zhang, 2013; Su et al., 2017; Niu et al., 2020)
Soft tissue	Tri, (Jafarzadeh et al., 2021) 4-node Tet, (Zhang et al., 2017; Chen et al., 2015; Peng et al., 2022; Hu et al., 2023; Cracknell et al., 2025; Du et al., 2024; Guo et al., 2016; Peng et al., 2021; Hsu et al., 2008; Chen et al., 2003; Zhang et al., 2020a; Wang et al., 2025; Lin et al., 2025) 10-node Tet, (Tang et al., 2019; Zhou et al., 2017; Yang et al., 2022; Strayer et al., 2020) 20-node Tet, (Chanda and Unnikrishnan, 2018) Tet (Cheung and Zhang, 2008; Cheung and Zhang, 2005; Shaulian et al., 2021; Shaulian et al., 2022; Liu and Zhang, 2013; Su et al., 2017; Sarikhani et al., 2016; Niu et al., 2020)
Muscle	20-node solid, (Serrato-Pedrosa et al., 2024a; Serrato-Pedrosa et al., 2024b) Connector, (Cheung and Zhang, 2008; Liu and Zhang, 2013; Peng et al., 2021; Zhang et al., 2020a) Link (Zhou et al., 2017)
Gastrocnemius	4-node Tet (Zhang et al., 2017)
Skin	20-node solid, (Serrato-Pedrosa et al., 2024a; Serrato-Pedrosa et al., 2024b) 3-node Tri membrane, (Peng et al., 2022; Peng et al., 2021) 4-node Quad, (Luo et al., 2011) 4-node Tet, (Hsu et al., 2008; Ab Rashid et al., 2022) Tet (Shaulian et al., 2021; Shaulian et al., 2022; Su et al., 2017)
Flexor tendon	Connector (Chen et al., 2015)
Achilles tendon	4-node Tet, (Chen et al., 2015) Tet (Shaulian et al., 2021; Shaulian et al., 2022)
Fascia	Link, (Nouman et al., 2023; Nouman et al., 2021; Hsu et al., 2008) Truss, (Zhang et al., 2017; Cheung and Zhang, 2008; Cheung and Zhang, 2005; Li and Yang, 2024; Jafarzadeh et al., 2021; Liu and Zhang, 2013; Du et al., 2024; Guo et al., 2016; Zhang et al., 2018; Zhang et al., 2020a) Spring, (Yang et al., 2022; Niu et al., 2020) 2-node Cable, (Chen et al., 2003) 4-node Tet, (Chen et al., 2015; Peng et al., 2022; Hu et al., 2023; Peng et al., 2021) Tet (Su et al., 2017)
Ligament	Link, (Zhou et al., 2017; Peng et al., 2022) Truss, (Zhang et al., 2017; Cheung and Zhang, 2008; Cheung and Zhang, 2005; Wang et al., 2023; Hu et al., 2023; Li and Yang, 2024; Jafarzadeh et al., 2021; Liu and Zhang, 2013; Du et al., 2024; Guo et al., 2016; Peng et al., 2021; Su et al., 2017; Zhang et al., 2018; Zhang et al., 2020a; Wang et al., 2025; Lin et al., 2025) 3-node Truss, (Peng et al., 2022) Spring, (Chen et al., 2015; Ab Rashid et al., 2022; Yang et al., 2022; Niu et al., 2020) 2-node Cable (Chen et al., 2003)
Meniscus	4-node Tet, (Zhang et al., 2017) 10-node Tet, (Zhou et al., 2017) Tet (Liu and Zhang, 2013)
Insole	high-order, (Serrato-Pedrosa et al., 2024a; Serrato-Pedrosa et al., 2024b) 4-node Quad, (Luo et al., 2011) 4-node Tet, (Nouman et al., 2021; Peng et al., 2022; Hu et al., 2023; Li and Yang, 2024; Cracknell et al., 2025; Guo et al., 2016; Peng et al., 2021; Hsu et al., 2008; Ab Rashid et al., 2022; Wang et al., 2025; Lin et al., 2025) 10-node Tet, (Geiger et al., 2023; Zhou et al., 2017; Yang et al., 2022; Strayer et al., 2020) 20-node Tet, (Chanda and Unnikrishnan, 2018) Tet, (Shaulian et al., 2021; Shaulian et al., 2022; Alsaleh et al., 2025; Sarikhani et al., 2016) Hex, (Cheung and Zhang, 2008; Cheung and Zhang, 2005; Jafarzadeh et al., 2021; Liu and Zhang, 2013; Su et al., 2017) 8-node Hex, (Zhang et al., 2017; Chen et al., 2015) 6-node Wedge (Chen et al., 2003), Quadratic element (Mala and Bisheh, 2025)
Sole	4-node Tet (Peng et al., 2022; Geiger et al., 2023)
Midsole	4-node Tet, (Peng et al., 2021; Ab Rashid et al., 2022) 10-node Tet, (Yang et al., 2022) Hex, (Cheung and Zhang, 2008) 8-node Hex (Chen et al., 2015)
Outsole	4-node Tet, (Ab Rashid et al., 2022) 10-node Tet, (Yang et al., 2022) Hex (Cheung and Zhang, 2008)
Upper	10-node Tet (Yang et al., 2022)
Heel cup	10-node Tet (Yang et al., 2022)
Ground	20-node solid, (Serrato-Pedrosa et al., 2024a; Serrato-Pedrosa et al., 2024b) 4-node Quad, (Luo et al., 2011) 4-node Tet, (Nouman et al., 2021; Hu et al., 2023; Hsu et al., 2008) Hex, (Cheung and Zhang, 2008; Jafarzadeh et al., 2021; Peng et al., 2021; Yang et al., 2022; Niu et al., 2020; Wang et al., 2025) 8-node Hex (Zhang et al., 2017; Peng et al., 2022; Li and Yang, 2024; Du et al., 2024; Lin et al., 2025)
Prosthesis	Tet (Guo et al., 2016)

Most studies did not report mesh density explicitly. A relatively complete foot model has been suggested to require at least 36,000 elements (Luboz et al., 2014), whereas more complex models can contain 400,000 elements (Chen et al., 2015). No gold standard exists for element type or mesh density in foot and footwear models (Chen et al., 2021). Mesh selection should therefore reflect the research question, model complexity and computational resources, with a documented balance between accuracy and efficiency.

### Material properties

In this review, the material property parameters from the included literature were extracted and systematically consolidated, with detailed data provided in Supplementary Appendix 4, although biological tissues generally exhibit complex nonlinearly behaviour, foot tissue properties are simplified by a linear elastic approximation in most studies (Song et al., 2022). The linear elastic properties assigned to bone are primarily based on a compromise between cortical and trabecular bone, resulting in a modulus of 7300 MPa and a Poisson’s ratio of 0.3 (Nakamura et al., 1981). This review, however, revealed that Nakamura’s report did not provide detailed data. In two studies (Guo et al., 2016; Goske et al., 2006), bone was simplified as a rigid structure, as the structural geometry of bone was considered to be more important than its actual mechanical properties. The linear elastic modulus values of the soft tissue ranged from 0.15–1.19 MPa, with Poisson’s ratios ranging from 0.45–0.49. Since plantar pressure is considered a key indicator for evaluating insole performance, soft tissue is modelled with more realistic isotropic and incompressible hyperelastic materials in most studies. This property is primarily derived from *in vivo* ultrasound measurements of plantar soft tissue stress-strain curves, expressed as second-order polynomial strain energy potentials (Lemmon et al., 1997). Alternative constitutive models, including the first-order Ogden (Chen et al., 2012) and Mooney-Rivlin models (Lemmon et al., 1997), were also used to describe hyperelasticity, whereas the Neo-Hookean and Prony models have been used to represent elastic and viscous responses (Levy et al., 2015; Katzengold and Gefen, 2018), respectively. Previous studies have indicated that plantar soft tissue is stiffer in patients with diabetes than in healthy individuals (Klaesner et al., 2002; Zheng et al., 2000), with 1.3–3 times greater stiffness observed in these patients. Accordingly, the elastic modulus of healthy soft tissue was multiplied by the above factors to represent stiffened soft tissue in five studies (Cao et al., 2021; Wang et al., 2023; Hu et al., 2023; Zhu et al., 2021; Niu et al., 2020). The linear elastic modulus of the cartilage, primarily derived from indentation device testing of the first metatarsophalangeal joint (Athanasiou et al., 1998), was mainly 1–10 MPa, with a Poisson’s ratio of 0.4. For the knee meniscus, the commonly reported modulus is 59 MPa, with a Poisson’s ratio of 0.49. Ligaments are almost universally simplified as linearly elastic materials, with values primarily determined from *in vitro* tensile tests of ankle collateral ligaments (Siegler et al., 1988). The modulus is typically 260 MPa, with a Poisson’s ratio of 0.4 and a cross-sectional area of 18.4 mm^2^. In only one study (Liu and Zhang, 2013), ligaments were modelled as hyperelastic, and the Mooney-Rivlin model was employed for knee ligaments. The linear elastic modulus of the plantar fascia was primarily determined on the basis of load-displacement or stress-strain curves obtained from *ex-vivo* and *in-vivo* plantar fascia tests (Wright and Rennels, 1964). The most frequently used modulus is 350 MPa, with Poisson’s ratios of 0.35–0.45 and a cross-sectional area of 58.6 or 290.7 mm^2^. With respect to the ligaments and fascia modelled as spring elements (Ab Rashid et al., 2022; Yang et al., 2022; Niu et al., 2020), the k values ranged from 39.1–1500 N/mm. To simplify the calculations, the ligaments and fascia are typically assigned the same overall modulus, with values assigned on the basis of the ligament type in only one study (Ab Rashid et al., 2022). Skin exhibits linear elasticity with a modulus range of 0.2–1.15 MPa and a Poisson’s ratio of 0.4–0.49. Its viscoelastic behaviour is described by the Prony and Neo-Hookean models (Levy et al., 2015; Katzengold and Gefen, 2018), whereas its hyperelasticity is modelled using the first-order Ogden (Pailler-Mattei et al., 2008) and Jamus-Green-Simpson models (Manschot and Brakkee, 1986). Muscles are uniformly simplified as linearly elastic with a modulus of 0.06–1.15 MPa and Poisson’s ratios of 0.49–0.495. Tendons have modulus values of 0.173–816 MPa and Poisson’s ratios of 0.3–0.49 (Kuo et al., 2001).

Some studies modelled insoles as linear elastic materials, with modulus values concentrated at 1–11.7 MPa or at 1000 MPa and above. The latter range is unlikely to reflect the stiffness of many insole materials and may reduce agreement between simulated and real-world behaviour. TPU was commonly assigned a modulus of 11–11.7 MPa and a Poisson’s ratio of 0.45, and porous units consistently used TPU. EVA, polyethylene and polyurethane were predominantly modelled using first- or second-order Ogden formulations. First- and second-order Ogden, Mooney-Rivlin, Neo-Hookean and Veronda-Westmann models were used to represent the nonlinear responses of insole materials under different loading states. However, unlike biological tissues, the mechanical properties of insole materials are highly dependent on material formulation, density, pore structure, and manufacturing processes. Therefore, material parameters reported in previous studies may not be directly applicable to the actual materials investigated, and obtaining the corresponding constitutive parameters through mechanical testing may be more appropriate. In addition, the foams commonly used in insoles exhibit pronounced strain-rate dependence, with their macroscopic mechanical response influenced by cell-structure deformation, polymer matrix properties, and pore-gas pressure or airflow. Previous studies have shown that the mechanical properties of polymeric foams vary with strain rate (Chan and Ngan, 2010). Therefore, under dynamic loading conditions such as walking, running, and landing, material parameters obtained from static or quasi-static tests may not fully represent the actual mechanical response of insole materials. However, few of the finite element studies included in this review have systematically considered the strain-rate dependence and dynamic mechanical behavior of insole materials.

### Loading and boundary conditions

Ground reaction forces (GRF) and Achilles tendon forces (ATF) were commonly applied and scaled to body weight. For static standing, GRF was often set to 50% of body weight on a single foot, whereas ATF was set to 25% or 37.5% of body weight (Cheung et al., 2006). Muscle-force-driven models can improve physiological realism, but were used infrequently (Cheung and Zhang, 2008; Chen et al., 2015; Peng et al., 2022; Wang et al., 2023; Liu and Zhang, 2013; Peng et al., 2021; Zhang et al., 2020a). Muscle forces were derived either from physiological muscle characteristics, combining physiological cross-sectional area with normalised electromyography during gait, or from integrated three-dimensional gait analysis and musculoskeletal multibody dynamics. Both approaches require additional experimental input and modelling assumptions, which may partly explain their limited adoption in existing insole-focused finite element studies.

In terms of the modes of loading conditions, point force is the most commonly adopted method in existing studies, in which external forces are applied to selected nodal features on the model surface or to a reference point coupled with specific model regions (Nouman et al., 2023; Nouman et al., 2021; Peng et al., 2022; Cao et al., 2021; Geiger et al., 2023; Wang et al., 2023; Hu et al., 2023; Zhu et al., 2021; Li and Yang, 2024; Jafarzadeh et al., 2021; Du et al., 2024; Peng et al., 2021; Ab Rashid et al., 2022; Yang et al., 2022). Alternative modes include displacement loading (Shaulian et al., 2021; Shaulian et al., 2022; Serrato-Pedrosa et al., 2024a; Serrato-Pedrosa et al., 2024b; Chen et al., 2003; Strayer et al., 2020) and contact loading (Chanda and Unnikrishnan, 2018; Mala and Bisheh, 2025). In displacement loading, the model motion is prescribed through defined displacement gradients, whereas in contact loading, the measured plantar pressure data are directly applied to the model surface. With respect to boundary conditions, the tibia, fibula, and upper surfaces of soft tissues at their proximal plane are typically constrained (Cheung and Zhang, 2008; Cheung and Zhang, 2005; Chen et al., 2015; Nouman et al., 2023; Nouman et al., 2021; Peng et al., 2022; Shaulian et al., 2021; Shaulian et al., 2022; Wang et al., 2023; Hu et al., 2023; Li and Yang, 2024; Jafarzadeh et al., 2021; Cracknell et al., 2025; Du et al., 2024; Peng et al., 2021; Su et al., 2017; Alsaleh et al., 2025; Serrato-Pedrosa et al., 2024a; Serrato-Pedrosa et al., 2024b; Hsu et al., 2008; Zhang et al., 2018; Zhang et al., 2020a; Zhang et al., 2020b; Yang et al., 2022; Niu et al., 2020; Wang et al., 2025; Lin et al., 2025). Some studies choose to fix the inferior surface of the insole (Chanda et al., 2018; Mala et al., 2025; Zhang et al., 2025; Zhou et al., 2017; Strayer et al., 2020) or the bottom surface located at the lowest part of the model (Geiger et al., 2023; Ab Rashid et al., 2022). Bonded or binding contacts are typically defined between various internal structures of the foot and footwear (Nouman et al., 2023; Nouman et al., 2021; Cao et al., 2021; Chanda and Unnikrishnan, 2018; Wang et al., 2023; Hu et al., 2023; Zhu et al., 2021; Zhang et al., 2025; Du et al., 2024; Goske et al., 2006; Zhang et al., 2020b; Ab Rashid et al., 2022; Yang et al., 2022; Lin et al., 2025), while frictional contact (with a coefficient of 0.3–0.6) is also defined between the overall foot, footwear, and ground (Zhang et al., 2017; Cheung and Zhang, 2008; Cheung and Zhang, 2005; Chen et al., 2015; Nouman et al., 2023; Nouman et al., 2021; Cao et al., 2021; Shaulian et al., 2021; Geiger et al., 2023; Shaulian et al., 2022; Luo et al., 2011; Wang et al., 2023; Hu et al., 2023; Li and Yang, 2024; Jafarzadeh et al., 2021; Tang et al., 2019; Zhang et al., 2025; Cracknell et al., 2025; Liu and Zhang, 2013; Du et al., 2024; Guo et al., 2016; Alsaleh et al., 2025; Serrato-Pedrosa et al., 2024a; Serrato-Pedrosa et al., 2024b; Hsu et al., 2008; Sarikhani et al., 2016; Goske et al., 2006; Chen et al., 2003; Zhang et al., 2018; Zhang et al., 2020a; Zhang et al., 2020b; Yang et al., 2022; Niu et al., 2020; Strayer et al., 2020; Wang et al., 2025). In addition, elastic plates may be bonded to rigid plates to simulate the ground (Peng et al., 2022; Peng et al., 2021).

Most studies simulated balanced standing or phases of walking stance, including heel strike, loading response, midstance, heel rise and pre-swing. However, real-life movements are far more complex and variable. Examples include heel strike during running (Yang et al., 2022) and single-leg landing (Ab Rashid et al., 2022) after a jump smash in badminton are receiving increasing simulation interests. Future studies should evaluate how insole parameters affect lower-limb biomechanics across a wider range of movements.

### Model validation and experimental validation

Foot models were commonly validated by comparing simulated and measured plantar-pressure distributions and peak values from the same gait trials. Complete validation of a numerical model remains unattainable, so some discrepancy between predictions and measurements is expected. In practice, errors within ± 5% are often considered acceptable. Discrepancies may result from physiological variation, simplified geometry or loading, and differences in spatial resolution between pressure-sensing systems and finite element meshes (Cheung and Zhang, 2005). Validation of internal tissue measures typically requires invasive or cadaveric experiments (Serrato-Pedrosa et al., 2024b), which are technically challenging and difficult to implement. Therefore, finite element studies focusing primarily on internal tissue measures rarely perform direct experimental validation. Some studies provide support for model predictions by conducting indirect comparisons with experimental data reported in existing literature (bone strain (Chen et al., 2015; Ab Rashid et al., 2022), joint contact pressure (Liu and Zhang, 2013), stress (Zhou et al., 2017), contact area (Liu and Zhang, 2013; Zhou et al., 2017), and muscle force (Peng et al., 2021)). However, such indirect comparisons generally provide more limited support for model reliability than direct comparisons with experimental data (Brown, 2004). Therefore, the results of finite element predictions for internal tissue measures should be interpreted with caution, particularly when direct experimental validation is unavailable. Nevertheless, this does not diminish the role of finite element analysis in reducing the potential harm to humans associated with *in vivo* studies, or its ability to assess the directional effects of a given design on specific biomechanical outcomes.

Current finite element studies on insoles possess several limitations. First, their demographic diversity and generalisability are limited: all models were based on a single participant; only four studies used female foot models (Zhang et al., 2017; Wang et al., 2023; Alsaleh et al., 2025; Zhang et al., 2018); and only seven studies reported participant nationality or ethnicity (Wang et al., 2023; Li and Yang, 2024; Du et al., 2024; Serrato-Pedrosa et al., 2024a; Serrato-Pedrosa et al., 2024b; Sarikhani et al., 2016; Ab Rashid et al., 2022). In intervention studies of pathological feet, models were often constructed from healthy feet, which may reduce clinical realism. Second, heterogeneity in study models and simulation conditions limits direct comparisons and evidence synthesis across studies, resulting in only limited evidence supporting many widely discussed conclusions. Finally, reporting of FEA methods was incomplete. Few studies described mesh sensitivity analysis in sufficient detail, mesh type and density were frequently unclear, FEA type was seldom specified, and experimental validation was rare.

### Future research

This synthesis identifies several priorities for future FEA research on insoles. First, the diversity of materials and lack of standard terminology hinder comparison across studies. Future reports should specify the material, product and manufacturer, together with relevant stiffness or elastic properties. Parameters derived from commercially available products or footwear standards may improve the interpretability of simulations and their relevance to clinical use. Second, most simulations do not represent real-world footwear conditions. Shoe characteristics, including toe-spring curvature and heel-to-toe drop, can influence lower-limb biomechanics (Zhu et al., 2024; Zhang et al., 2021; Stewart et al., 2007), yet many studies omit the upper and thus assume unrestricted dorsal-foot motion (Cheung and Zhang, 2005). This can produce unrealistic insole geometries that occupy excessive shoe volume and increase dorsal pressure, compromising comfort or safety. Third, plantar pressure remains a key outcome. The plantar and heel regions bear the highest loads, and 200 kPa has been proposed as a pressure threshold associated with ulcer risk (Cao et al., 2021; Wang et al., 2023; Jafarzadeh et al., 2021). Studies of diabetes or foot ulcers should therefore report whether simulated or measured pressures are reduced relative to this threshold. Fourth, future studies should, where appropriate, refer to model configurations and key simulation conditions used in previous studies to improve cross-study comparability. Key factors, including model configurations, material constitutive behavior, control group, and modeled participant characteristics, should be kept consistent where possible or based on clearly justified parameter settings. Comprehensive reporting and evaluation of relevant outcome measures should also be encouraged to facilitate comparisons and evidence synthesis across studies. Fifth, insole performance is multifactorial, whereas conventional mechanical testing becomes costly when many parameters and levels are examined. Taguchi designs can efficiently explore multiparameter combinations using fewer simulations and quantify the contribution of each parameter (Cheung and Zhang, 2008; Alsaleh et al., 2025; Yang et al., 2022; Ryan, 2005) Comparable optimisation frameworks should be evaluated to reduce the computational burden of FEA. Finally, graph neural networks can approximate foot FEA at substantially lower computational cost (Kang et al., 2025). Their use as surrogate models may accelerate the development and evaluation of personalised insoles.

### Limitation

This review has several limitations. First, we used best-evidence synthesis rather than meta-analysis, so we could not estimate pooled effect sizes or quantify overall trends precisely. Second, the search was restricted to Chinese- and English-language literature and did not use snowball sampling, which may have omitted relevant studies. Third, data extraction was deliberately conservative: information that was underreported or not explicitly reported was recorded as ‘Unknown’, even when aspects of the analysis might have been inferred from loading and boundary conditions. This approach was intended to improve transparency and consistency.

## Conclusion

Limited evidence indicates that insoles may have biomechanical benefits for foot. Conforming insoles were consistently associated with lower plantar pressure, although the optimal conformity index remains uncertain. Wedge and arch-support insoles may improve foot alignment, but their parameters should remain within appropriate ranges to avoid excessive loading of adjacent tissues. Low-modulus materials and local plug- or hole-structured designs may reduce pressure in high-load regions. Large single plugs or holes should be used cautiously because they may create secondary pressure peaks; edge stiffness gradients or multiple smaller structures may reduce this risk. Greater thickness and lower material stiffness may also attenuate plantar pressure, but excessive thickness can restrict shoe-cavity volume and contribute to toe-box constriction, whereas overly compliant insoles may bottom out because of inadequate support.

The design characteristics of non-featured, metatarsal, heel, and comprehensive insoles varied considerably, and the evidence supporting their effects was limited. No consensus has been reached regarding the optimal configurations of wedge, arch-support, multihole and multiplug designs. However, owing to limitations related to subject-specific models and insufficient experimental validation of internal tissue mechanical measures, the clinical therapeutic effects of insoles for specific conditions cannot currently be established on the basis of the available evidence. Therefore, future studies should conduct clinical intervention and follow-up studies in specific patient populations to further evaluate the actual effects of insole designs on disease-related clinical outcomes. Moreover, inappropriate simplification of finite element models may not only compromise the validity of the predicted results but also affect comparability across studies. Finally, inconsistent reporting of insole designs, difficulties in translating simulated concepts into manufacturable products and limited scalability also hinder synthesis and application. Addressing these limitations is necessary to move insole FEA from computational demonstration towards clinically and industrially applicable solutions.

## Data Availability

The original contributions presented in the study are included in the article/Supplementary Material, further inquiries can be directed to the corresponding authors.
