# Supplementary material for: Finite element analysis of foot-insole biomechanics for insole design: a systematic review
Source: Front Bioeng Biotechnol. 2026 Oct 28;14:1994273. doi: 10.3389/fbioe.2026.1994273 (PMC13750745; doi:10.3389/fbioe.2026.1994273)
Supplement: Supplementary file 1 [file Supplementaryfile1.docx]

| **Appendix 1 Data extraction table** | | | | | | | | | | | |
| --- | --- | --- | --- | --- | --- | --- | --- | --- | --- | --- | --- |
| **Authors** | **Research content** | **Model acquisition** | **Mesh type** | **Movement** | **Material properties** | **Loading conditions** | **Boundary conditions** | **Analysis type** | **Outcome indicators** | **Model validation** | **Experimental validation** |
| Alsaleh et al., 2025 | Arch support insole: arch height (30, 37, 42 mm), insole thickness (8, 10, 12 mm), heel cup height (16, 18, 20 mm), insole material (EVA, TPU, rubber) | Scanning, modeling | Tet | Walking (mid-stance) | Linearly elastic (insole: EVA, TPU, rubber), rigid (ground), unknown (foot) | Mechanical loading (GRF: 421.8N) | Constraint (the upper surfaces of tibia, fibula, and soft tissue), frictional contact (foot & insole: 0.5) | 3D, static | Plantar pressue, stress, strain and deformation | Unknown | Unknown |
| Cracknell et al., 2025 | Variable stiffness insole: an insole adjusting porous parameters (wall thickness, cell size, and aspect ratio) for different stiffness ranges, with a fluid-filled condition | Scanning, modeling | 4-Node Tet (soft tissue, bone, insole) | Walking (heel strike, foot flat,  mid-stance, terminal stance, heel off, toe off) | Linearly elastic (bone), hyperelastic (soft tissue, insole: porous structure), rigid (ground), unknown (insole: fluid) | Mechanical loading (reaction force and moment) | Fix (the upper surfaces of ankle bones, ground: x-axis rotation only), tie constraint (bone & soft tissue), frictional contact (insole & foot: 0.6), radian (foot & ground, each stage of walking: 0, 0.123, 0.175, 0.187, 0.62, 0.815 °) | 3D, static | Plantar pressue | Plantar pressue | Plantar pressue |
| Lin et al., 2025 | Nonfeatured insole: insole stiffness (100, 260, 1000, 3000, 5000, 500000 MPa) | CT | 4-node Tet (cartilage, cortical bone, cancellous bone, insole, soft tissue), truss (ligament), 8-node Hex (ground) | Standing and loading phase of gait | Linearly elastic (cortical bone, cancellous bone, cartilage, ligament, soft tissue, ground), unknown (insole) | Mechanical loading (GRF: 300N, ATF: 150N) | Constraint (the upper surfaces of tibia and fibula), bonded contact (ligament & bone, foot & insole), frictional contact (insole & ground: 0.6), node-to-node contact & no-separation contact (bone & cartilage) | 3D, static | Foot deformation, bone tuberosity stress, metatarsal stress, plantar stress | Plantar pressue | Unknown |
| Mala et al., 2025 | Hole-structured insole: hole shape (none, circular hole, irregular hole), insole material (EVA: Shore A-65, 85, 95 and PU) | Modeling | Quadratic element (foot, insole) | Standing | Linearly elastic (DM foot), unknown (insole: EVA, PU) | Contact loading (load applied on the top surface of the foot: 0.3 MPa) | Constraint (the bottom of the insole and side edge) | 3D, static | Plantar stress | Plantar stress | Unknown |
| Wang et al., 2025 | Nonfeatured insole: an insole with a metatarsal pad, a toe pad, and arch support | CT | Truss (ligament), Hex (ground), 4-Node Tet (bone, soft tissue, insole) | Standing | Linearly elastic (all) | Mechanical loading (BWF: 325N, ATF: 243.75N) | Fix (the upper surfaces of tibia, fibula, and soft tissue), frictionless and hard contact (cartilage & bone), frictional contact (foot & insole: 0.5, insole & ground: 0.6) | 3D, static | Fascia stress, bone stress | Plantar pressue | Unknown |
| Zhang et al., 2025 | Variable stiffness insole: an insole with an auxetic structure, stiffness (first layer: 1.7 MPa, second layer: 3.08 MPa, third layer: 11.7 MPa, fourth layer: 11.7 MPa) | Unknown | 4-Node Tet (heel pad) | Walking (mid-stance) | Hyperelastic (skin, heel pad), unknown (bone, muscle, cartilage, ground, insole) | Unknown | Fix (the lower surface of insole), bonded contact (tissue layers), frictional contact (foot & insole) | 3D, unknown | Soft tissue stress, total stress concentration exposure of soft tissue, heel pad stress, skin stress | Plantar pressue | Unknown |
| Serrato-Pedrosa et al., 2024a | Conforming insole (full conforming) | CT, modeling | 20-node solid (skin, muscle, ground), high-order (insole) | Standing | Linearly elastic (skin, muscle), unknown (ground, insole: TPU) | Displacement loading (ground: vertical displacement 5 mm to assume 400 N GRF) | Constraint (the upper area of soft tissue, forefoot, instep, toe, and around the foot), frictional contact (foot & insole: 0.5, foot & plate: 0.5) | 3D, static | Plantar deformation, plantar stress | Plantar pressure | Unknown |
| Serrato-Pedrosa et al., 2024b | Conforming insole (full conforming) | CT, modeling | 20-node solid (skin, muscle, ground), high-order (insole) | Walking (heel strike, loading response, mid-stance, heel rise, toe off) | Linearly elastic (skin, muscle, insole: TPU), rigid (ground) | Displacement loading (ground: vertical displacement 6, 7, 5.5, 8, 8.5 mm to assume GRF) | Constraint (the upper, medial-lateral, instep, and toe areas of soft tissue), Frictional contact (foot & insole: 0.5), angle (0 °, heel lift angle: 14, 8 °, forefoot lift angle: 18, 30 °) | 3D, quasi-  static | Plantar stress, insole stress | Plantar pressure | Unknown |
| Li et al., 2024 | Variable stiffness insole: thickness (3-10 mm), stiffness (1.04-74 MPa) | CT, modeling | 4-node Tet (bone, test insole, insole), truss (fascia, ligament), 8-node Hex (ground) | Standing | Linearly elastic (bone, ligament, fascia, test insole, insole, ground), hyperelastic (soft tissue) | Mechanical loading (GRF: 305 N, ATF: 150 N) | Constraint (the upper surfaces of tibia, fibula, and soft tissue, ground: vertical movement only), frictional contact (foot & ground: 0.6) | 3D, static | plantar contact area, plantar pressure, soft tissue stress | plantar pressure | Unknown |
| Du et al., 2024 | Wedge insole: lateral wedge angle (0, 5, 7 °) | CT | 4-node Tet (bone, soft tissue), truss (ligament, fascia), 8-node Hex (ground) | Walking (the first peak of vertical GRF, GRF valley, the second peak of vertical GRF) | Linearly elastic (bone, cartilage, ligament, fascia, soft tissue), unknown (insole: EVA, the upper layer of ground), rigid (the lower layer of ground) | Mechanical loading (GRF) | Constraint (the upper surfaces of tibia, fibula, and soft tissue), frictionaless contact (joint & joint), bonded contact (ligament & bone, soft tissue & bone), frictional contact (foot & insole: 0.1, insole & ground: 0.6) | 3D, static | Plantar pressure, joint contact pressure, bone stress, ligament stress | Plantar pressure | Unknown |
| Geiger et al., 2023 | Plug-structured insole: plug stiffness (0.5, 1.0, 1.5 MPa) | MRI, scanning, modeling | 10-node Tet (foot, insole), 4-node Tet (sole) | Walking (the early and late middle stance) | Hyperelastic (soft tissue), linearly elastic (bone, insole: TPC, sole), unknown (plug) | Displacement loading (talus: vertical displacement 10 mm, with constraint on translation and rotation), mechanical loading (JRF and JM, with constraint on x-axis rotation) | Fix (the lower surface of sole), tie contact (insole & sole), frictional contact (foot & insole: 0.5) | 3D, quasi-static | Plantar pressure | Plantar pressure | Unknown |
| Nouman et al., 2023 | Nonfeatured insole: insole type (heel insole, heel-forefoot insole), insole material (Plastazote PE, Nora Lunalastike, EVA, firm, void) | CT, modeling | Tet (foot), link (fascia) | Standing | Linearly elastic (bone, soft tissue, fascia, insole: Plastazote PE, Nora Lunalastike, EVA, TPU, firm, ground) | Mechanical loading (GRF: 420 N, ATF) | Fix (the upper surface of tibia and fibula), bonded contact (bone & soft tissue), frictional contact (foot & insole: 0.3, insole & ground: 0.6) | 3D, static | Plantar pressure, plantar frictional stress | Unknown | Unknown |
| Wang et al., 2023 | Variable stiffness insole: the wall thickness of porous units (0.8, 1.0, 1.2, 1.4 mm, equivalent to 1.9, 2.5, 3.1, 3.6 MPa), corresponds to three variable stiffness insole schemes | CT, MRI | Truss (ligament) | Walking (heel strike, mid-stance, toe off) | Linearly elastic (cartilage, fascia, bone, ground), hyperelastic (healthy soft tissue, DM soft tissue, test insole: EVA), unknown (ligament, insole: TPU) | Mechanical loading (GRF: 775 N, 680 N, 775 N, ATF: 1300 N, 532 N, 76 N) | Constraint (the upper surface of lower leg, ground: vertical movement only), bonded contact (insole & ground), frictional contact (foot & insole: 0.6), angle (lower leg & coronal plane: 18, 9, 2 °) | 3D, static | Plantar pressure, soft tissue stress, cartilage stress | Plantar pressure | Unknown |
| Hu et al., 2023 | Variable stiffness insole: the wall thickness of porous units (1.2, 1.4, 1.6, 2.0 mm, equivalent to 0.47, 0.95, 1.39, 1.9, 2.47 MPa), corresponds to five variable stiffness insole schemes | CT | 4-node Tet (fascia, bone, cartilage, insole, soft tissue, ground), truss (ligament) | Standing | Linearly elastic (bone, cartilage, fascia, ligament, insole: TPU, ground), hyperelastic (soft tissue, DM soft tissue) | Mechanical loading (GRF: 380 N, ATF: 285 N) | Constraint (the upper surface of tibia, fibula, and soft tissue, ground: vertical movement only), frictional contact (foot & insole: 0.6), bonded contact (insole & ground) | 3D, static | Plantar pressure, soft tissue stress, bone stress | Plantar pressure | Unknown |
| Shaulian et al., 2022 | Hole-structured/plug-structured insole: insole conforming index (flat, full conforming), insole configuration (hole insole, hole and fill insole, graded stiffness insole, graded stiffness with hole insole), graded stiffness material gradient transition method | CT, modeling | Tet (all) | Walking (heel strike) | Linearly elastic (bone, cartilage, achilles tendon), visco-hyperelastic (skin, soft issue), hyperelastic (insole & plug & insole cover & graded material: EVA) | Displacement loading (insole: vertical displacement 7-10 mm to assume 320N GRF) | Constraint (the upper surfaces of tibia, fibula, and soft tissue), frictional contact (foot & insole: 0.6), angle (forefoot lift angle: 10 °) | 3D, quasi-static | Soft tissue stress, total stress concentration exposure of soft tissue | Unknown | Unknown |
| AB Rashid et al., 2022 | Nonfeatured insole: insole shape (Yonex Active Pro Truactive, Victor VT-XD 8, Li-Ning L6200LA) | CT, scanning, modeling | 4-node Tet (bone, skin, insole, midsole, outsole), spring (ligament) | Single-leg landing | Linearly elastic (bone, skin, insole), unknown (ligament, midsole, outsole) | Mechanical loading (load applied on the skin: 1927.5 N) | Fix (the lower surface of the outsole), bonded contact (all), angle (midfoot and hindfoot lift angle: 2.95 °) | 3D, static | Insole stress, bone stress, bone displacement, bone rotation angle | Bone strain, plantar pressure | Unknown |
| Yang et al., 2022 | Nonfeatured insole: insole material (EVA, latex), insole thickness (0, 3, 6 mm), heel cup conforming index (flat, half conforming, full conforming), midsole material (Asker C-45, 60, 70) | CT, modeling | 10-node Tet (Soft tissue, bone, cartilage, upper, outsole, midsole, insole, heel cup), Hex (ground), spring (ligament, fascia) | Running (heel strike) | Unknown (soft tissue, bone, cartilage, fascia, ground, upper, outsole: rubber), hyperelastic (insole & heel cup: EVA/latex, midsole: EVA) | Mechanical loading (ATF: 186 N, NAJF: 802 N, ankle dorsiflexion moment: 10 N/m), displacement loading (ground: vertical displacement to assume 1149 N GRF) | Fix (the upper surfaces of tibia and fibula, ground: vertical movement only), bonded contact (bone & soft tissue & cartilage, insole & midsole, midsole & outsole), frictional contact (bone & soft tissue & cartilage, foot & insole, upper, outsole & ground: 0.6), remote displacement (tongue loop), angle (foot strike angle: 14 °) | 3D, quasi-static | Plantar pressure | Plantar pressure, outsole pressure | Unknown |
| Peng et al., 2022 | Arch support insole/wedge insole (multi-parameter design): arch support height (42, 45, 48 mm), medial wedge angle (0, 2, 4 °), heel cup height (14, 16, 18 mm), insole stiffness (3, 5, 7 MPa) | MRI, modeling | 4-node Tet (bone, soft tissue, insole, shoe, fascia), 8-node Hex (ground), 3-node Tri membrane (skin), 3-node truss (ligament) | Walking (mid-stance) | Hyperelastic (skin, soft tissue), unknown (bone, ligament, fascia, midsole, insole, the upper layer of ground), rigid (the lower layer of ground) | Mechanical loading (GRF: vertical force 338 N, mediolateral force 6 N, anteroposterior force 4 N, ATF: 774 N, muscles force: TAF: 38 N, TPF: 200 N, PBF: 1 N, PLF: 0 N, FHLF: 95 N, FDLF: 26 N) | Fix (the proximal surfaces of fibula, tibia, and soft tissue), frictionless contact (joint & joint), tie contact (ground & rigid plate) | 3D, static | Fascia strain, plantar pressure | Plantar pressure | Unknown |
| Peng et al., 2021 | Arch support insole: arch support height (42, 45, 48 mm) | MRI, scanning, modeling | Hex (ground), 4-node Tet (bone, soft tissue, insole, midsole, fascia), 3-node Tri membrane (skin), truss (ligament), connector (muscle) | Walking (the first peak of vertical GRF, GRF valley, the second peak of vertical GRF) | Hyperelastic (skin, soft tissue), unknown (bone, ligament, fascia, insole: TPU), rigid (the lower layer of ground) | Mechanical loading (GRF, ATF, muscle force: TAF, TPF, PBF, PLF, FHLF, FDLF) | Fix (the proximal surfaces of tibia, fibula, and skin), tie contact (bone & soft tissue, fascia & soft tissue, calcaneal tubercle, and proximal phalange, ground & rigid plate), frictionless contact (bone & bone) | 3D, static | Plantar pressure, fascia strain, ligament stress, arch height | Plantar pressure, muscle force | Unknown |
| Jafarzadeh et al., 2021 | Variable stiffness insole: variable stiffness (0.4-74 MPa), variable shape | CT, modeling | Tet (bone), truss (ligament, fascia), Hex (insole, ground), Tri (soft tissue) | Standing | Linearly elastic (bone, ligament, fascia, ground, insole: initial & EVA), hyperelastic (soft tissue) | Mechanical loading (GRF: 395 N, ATF) | Fix (the upper surfaces of soft tissues, tibia, and fibula), surface to surface contact (foot & insole), Frictionless contact (bone & cartilage) | 3D, static | Plantar pressure | Unknown | Unknown |
| Zhu et al., 2021 | Variable stiffness insole: the wall thickness of porous units (0.6, 1.0, 1.4, 1.8 mm) | Scanning, database, modeling, CT | Unknown | Standing | Linearly elastic (bone, soft tissue, DM soft tissue), unknown (test insole, insole: TPU, ground) | Mechanical loading (GRF, ATF) | Fix (the upper surface of talus), bonded contact (bone & soft tissue) | 3D, static | Plantar contact pressure, soft tissue stress | Plantar contact pressure, plantar contact area, vertical compression of soft tissue, soft tissue stress | Unknown |
| Cao et al., 2021 | Hole-structured insole: hole interval (2, 3, 4 mm), hole depth (2, 4, 6 mm), hole diameter (3, 4, 5 mm) | CT | Unknown | Standing | Linearly elastic (bone, ground), hyperelastic (healthy soft tissue, DM soft tissue, insole: Poron_L24) | Mechanical loading (upward load applied on the calcaneus and ground) | Fix (the upper surface of talus, ground: vertical movement only), bonded contact (bone & soft tissue), frictional contact (foot & insole & ground: 0.6) | 3D, static | Plantar pressure, soft tissue stress | Plantar pressure | Plantar pressure |
| Nouman et al., 2021 | Nonfeatured insole: the materials of single-layer insole (EVA, Amfit, TPU), the material of double-layer insole (EVA & TPU, Amfit & TPU) | CT, modeling | 4-node Tet (foot, insole, ground), link (fascia) | Standing | Linearly elastic (bone, soft tissue), unknown (fascia, insole: EVA, Amfit, TPU, ground) | Mechanical loading (GRF: 420 N, ATF) | Fix (the upper surface of tibia and fibula), bonded contact (bone & soft tissue), frictional contact (foot & insole, insole & ground: 0.3) | 3D, static | Plantar pressure, plantar frictional stress | Plantar pressure | Unknown |
| Shaulian et al., 2021 | Hole-structured insole: hole radius (5-30 mm), the radius of curvature of the edges (2-230 mm), hole depth (1-15 mm) | CT, modeling | Tet (all) | Walking (heel strike) | Linearly elastic (bone, achilles tendon), visco-hyperelastic (skin, soft tissue), hyperelastic (insole: EVA), unknown (cartilage) | Displacement loading (insole: upward displacement 7-10 mm to assume 320 N GRF) | Fix (the upper surfaces of tibia, fibula and soft tissues), frictional contact (foot & insole: 0.6), angle (forefoot lift angle: 10 °) | 3D, static | Soft tissue stress, total stress concentration exposure of soft tissue | Plantar pressure | Unknown |
| Zhang et al., 2020b | Arch support insole/wedge insole (multi-parameter design): arch support height (20, 25, 30 mm), lateral wedge angle (3, 5, 7 °), insole stiffness (1.0, 1.5, 2.5 MPa) | CT, modeling | Solid, truss | Walking (mid-stance) | Linearly elastic (bone, cartilage, fascia, ligament), hyperelastic (soft tissue), unknown (ground, insole, test insole) | Mechanical loading (GRF, ATF: 640 N) | Fix (the upper surfaces of tibia and fibula), bonded contact (cartilage & joint surface), frictional contact (foot & insole & ground: 0.6) | 3D, quasi-static | Plantar stress, soft tissue stress, cartilage stress, fascia stress | Plantar stress, plantar contact area | Unknown |
| Zhang et al., 2020a | Arch support insole/wedge insole (multi-parameter design): arch support height (20, 25, 30 mm), lateral wedge angle (3, 5, 7 °) , insole stiffness (1.0, 1.5, 2.5 MPa) | CT, modeling | 4-node Tet (bone, arthritic cartilage, soft tissue), truss (ligament, fascia), connector (muscle) | Walking (mid-stance) | Linearly elastic (bone), hyperelastic (soft tissue), unknown (arthritic cartilage, fascia, ligament, insole), rigid (ground) | Mechanical loading (GRF: 600 N, ATF: 640 N, muscle force: TPF: 50 N, PMGF: 50 N, FDLF: 36 N, FHLF: 20 N) | Constraint (the upper surfaces of tibia, fibula and soft tissue), frictional contact (foot & insole, insole & ground: 0.6) | 3D, static | Cartilage stress, plantar pressure, bone stress | Plantar pressure, plantar contact area | Unknown |
| Niu et al., 2020 | Nonfeatured insole: a layered modular insole, with eight layers of small cushions, each with a thickness of 0.5 mm | CT | Tet (bone, soft tissue, cartilage), spring (ligament, fascia), Hex (ground) | Standing | Linearly elastic (bone, ligament, cartilage, fascia), hyperelastic (healthy soft tissue, DM soft tissue), rigid (ground) | Mechanical loading (GRF: 370 N) | Fix (the upper surfaces of tibia, fibula, and soft tissue), frictionless contact (joint surface & joint surface), frictional contact (foot & insole, insole & ground: 0.6) | 3D, static | Plantar pressure | Unknown | Unknown |
| Strayer et al., 2020 | Nonfeatured insole: an insole with pressurized bubbles, bubble height (4.9-10 mm), bubble stiffness (3, 5, 10 MPa) | Database, modeling | 10-node Tet (all) | Standing | Linearly elastic (bone, insole: PVC), hyperelastic (soft tissue) | Displacement loading (bone: negative y-axis displacement to assume 130 N GRF) | Constraint (the lower surface of insole, the upper surface of the bone: negative y-axis movement only), frictional contact (foot & insole: 0.6) | 3D, quasi-static | Plantar contact pressure | Plantar contact pressure | Unknown |
| Tang et al., 2019 | Variable stiffness insole: the wall thickness of porous units (0.6, 1.0, 1.4, 1.8 mm), the type of porous units | CT, modeling | 10-node Tet (bone, soft tissue) | Standing | Linearly elastic (bone, soft tissue), unknown (insole: TPU), rigid (ground) | Mechanical loading (GRF, ATF) | Constraint (the upper surface of talus, the distal surface of insole constrained to the ground), tie contact (bone & soft tissue), frictional contact (foot & insole: 0.3) | 3D, static | Plantar pressure, plantar contact area | Plantar pressure | Plantar pressure, contact area |
| Zhang et al., 2018 | Partial insole (heel insole): insole shape (circular, horizontal elliptical, vertical elliptical), insole thickness (2, 3, 4 mm), groove type (round hole, honeycomb, groove) | CT, modeling | Truss (ligament, fascia) | Standing | Linearly elastic (bone, soft tissue, ligament, fascia), unknown (insole: EVA, test insole), rigid (ground) | Mechanical loading (GRF: 320 N, upward load applied at the calcaneus-achilles tendon junction: 240 N) | Constraint (the upper surfaces of tibia and fibula), frictional contact (foot & insole & ground: 0.6) | 3D, static | Plantar stress, soft tissue stress, fascia stress | Plantar stress | Unknown |
| Chanda et al., 2018 | Hole-structured insole: an insole with arch support and ulcer isolation, insole stiffness (Veronda-Westmann, C_1_ = 1.3-25.4 MPa, C_2_ = 0.18-0.29 MPa) | Manual measure-ment, modeling | 20-node Tet (all) | Standing | Linearly elastic (DM foot), hyperelastic (insole) | Contact loading (plantar pressure: 0.3 MPa) | Constraint (the lower surface of insole), bonded contact (foot & insole) | 3D, static | Plantar stress | Plantar stress | Unknown |
| Zhang et al., 2017 | Conforming insole: conforming index (flat, half conforming, full conforming), insole thickness (4, 5, 6, 7 mm), insole stiffness (0.5, 1.0, 1.5, 2.0 MPa) | CT, modeling | 8-node Hex (ground, insole), 4-node Tet (bone, soft tissue, gastrocnemius, meniscus), truss (ligament, fascia) | Standing | Linearly elastic (bone, soft tissue, fascia, ligament, gastrocnemius, meniscus, ground, insole: PU & EVA) | Mechanical loading (upward load applied on the ground: 320 N, muscle force: gastrocnemius force: 240 N) | Constraint (the upper surface of femur), bonded contact (insole & ground), frictionless contact (tibia & femur & meniscus), frictional contact (foot & insole: 0.6) | 3D, static | Plantar pressure, metatarsal stress | Plantar pressure | Unknown |
| Zhou et al., 2017 | Wedge insole: lateral wedge angle (0, 4, 8, 12, 16 °) | CT | 10-node Tet (bone, soft tissue, cartilage, meniscus, insole), link (ligament, muscle) | Walking (mid-stance) | Linearly elastic (bone, cartilage), unknown (soft tissue, meniscus, ligament, insole) | Mechanical loading (BWF: 1000 N, muscle force: gluteus medius abductor force: 500 N) | Constraint (the lower surface of insole, femur & tibia & fibula: with constraint x and y-axis displacement), tie contact (bone & bone) | 3D, static | Subtalar joint contact area, subtalar joint stress | Subtalar joint contact area, subtalar joint stress | Unknown |
| Su et al., 2017 | Arch support insole: insole stiffness (Shore-A 30, 35, 40), arch support height (27, 30, 33 mm) | CT, scanning, modeling | Tet (bone, soft tissue, fascia, cartilage, skin), truss (ligament), Hex (ground) | Standing | Linearly elastic (bone, soft tissue, fascia, cartilage, ligament, skin), unknown (ground) | Mechanical loading (ATF: 375 N), displacement loading (navicular bone displacement: vertical displacement) | Fix (the upper surfaces of tibia, fibula and skin), frictionless contact (cartilage & cartilage), tie constraint (ligament, skin, and cartilage & bone) | 3D, quasi-static | Arch height, plantar pressure, cartilage stress, ligament stress | Plantar pressure | Unknown |
| Guo et al., 2016 | Wedge insole: medial wedge angle (0-10 °) | MRI, modeling | 4-node Tet (bone, insole, soft tissue), Tet (cartilage, prosthesis), truss (fascia, ligament) | Standing | Linearly elastic (bone, cartilage, ligament, fascia, prosthesis: silicon rubber), hyperelastic (soft tissue, insole: PU) | Mechanical loading (BWF: 320 N, ATF) | Constraint (the point of load application at the center of pressure on the distal tibia and fibula: vertical movement only), frictional contact (foot & insole: 0.6) | 3D, static | Bone stress, ligament tension, fascia stress, plantar pressure | Plantar pressure | Unknown |
| Sarikhani et al., 2016 | Conforming insole: conforming index (flat, full conforming), insole stiffness (2000, 1000, 0.3 MPa), full conforming insole thickness (0.25, 0.5, 0.75, 1, 2 cm), full conforming insole heel height (0.25, 0.5, 1 °), flat insole thickness (0.25, 0.5, 1 cm), flat insole heel height (0.25, 0.5, 1, 2 °) | CT, modeling | Tet (all) | Unknown | Linearly elastic (bone, insole), hyperelastic (soft tissue) | Mechanical loading (load applied on the achilles tendon) | Frictional contact (foot & insole: 0.6) | 3D, unknown | Planter stress | Planter stress | Unknown |
| Chen et al., 2015 | Partial insole (metatarsal insole): insole thickness (2.5, 5.1, 7.6, 10.2, 12.7 mm), insole position (none, distal: 6.5 mm proximal to the center of the second metatarsal head, proximal: 19.5 mm, most proximal: 26.5 mm) | Reference | 4-node Tet (bone, cartilage, fascia, achilles tendon, soft tissue), spring (ligament), 8-node Hex (insole, midsole), connector (flexor tendon) | Walking (The second peak of GRF) | Linearly elastic (ligament, cartilage), hyperelastic (insole: Plastazote, midsole: Microcell Puff, soft tissue), unknown (bone, fascia, achilles tendon, flexor tendon) | Mechanical loading (GRF: 623.1 N, muscle force: GSF: 1620 N, TPF: 267 N, FHLF: 130 N, FDLF: 81 N, PBF: 91 N, PLF: 193 N) | Constraint (tibia, fibula, midsole constrained to the ground), frictional contact (foot & insole: 0.5), angle (the second metatarsal bone & sagittal plane: 26.81 °, tibial inclination angle: 18.11 °) | 3D, static | Plantar pressure, soft tissue strain, bone stress, fascia tensile stress, toe pad compressive strain | Plantar pressure, bone strain | Unknown |
| Liu et al., 2013 | Wedge insole: lateral wedge angle (0, 5, 10 °) | MRI, modeling | Truss (ligament, fascia), Tet (bone, soft tissue, cartilage, meniscus), connector (muscle), Hex (insole) | Walking (single foot stance) | Linearly elastic (bone, menisci, cartilage), hyperelastic (soft tissue, ligament) | Mechanical loading (GRF, muscle force: QUADSF: 200 N, HAMF: 80 N, GASF: 300 N, SOLF: 400 N, TPF: 70 N, FHLF: 40 N, FDLF: 30 N, PBF: 30 N, PLF: 40 N) | Constraint (distal femur, tibia: 6 degrees freedom only), frictional contact (foot & insole: 0.6), frictionless contact (bone & bone, cartilage & meniscus), tie contact (ligament, muscle & bone) | 3D, quasi-  static | Cartilage stress, meniscus stress, meniscus contact force | Plantar pressure, plantar contact area, joint contact pressure, joint contact area | Unknown |
| Luo et al., 2011 | Hole-structured insole: hole-structure configuration (cylindrical: 14 or 20 mm diameter, conical: top or bottom 2.5 mm and 25 mm base diameter), conforming index (flat, full conforming), insole stiffness (same as heel pad, 2.5 times, 6 times) | MRI | 4-node Quad (all) | Standing | Hyperelastic (skin, heel pad, insole), linearly elastic (ground), rigid (bone) | Mechanical loading (BWF: 200 N) | Constraint (calcaneus), frictionless contact (bone & heel pad), glued (heel pad & skin, bone & heel pad), frictional contact (foot & insole: 0/0.2, insole & ground: 0/0.3) | 2D, static | Stress, strain and strain energy density of heel pad | Plantar pressure | Unknown |
| Cheung et al., 2008 | Arch support insole: arch support shape (flat, full-weight-bearing, half-weight-bearing, non-weight-bearing), insole thickness (3, 6, 9, 12 mm), midsole thickness (3, 6, 9, 12 mm), insole stiffness (Shore-A 10, 20, 30, 40), midsole stiffness (Shore-A 20, 30, 40, 50) | MRI, scanning | Tet (bone, soft tissue, cartilage), truss (ligament, fascia), Hex (insole, midsole, outsole, ground), connector (muscle) | Walking (mid-stance) | Linearly elastic (bone, ligament, fascia, cartilage), hyperelastic (soft tissue, insole & midsole & outsole: PU & EVA), unknown (ground) | Mechanical loading (GRF: 550 N, ATF: 700 N, muscle force: TPF: 70 N, FHLF: 40 N, FDLF: 30 N, PBF: 30 N, PLF: 40 N, LRF: 50 N, MRF: 50 N) | Fix (sole fixed on the ground, the upper surface of soft tissue, the distal surfaces of tibia and fibula), frictional contact (foot & insole: 0.6) | 3D, static | Plantar pressure | Unknown | Plantar pressure |
| Actis et al., 2008 | Plug-structured insole: plug configuration (multi-plug, single-plug), single-plug type (partially penetrating, fully penetrating), multi-plug number (5-15), diameter (2-8 mm), interval (1-5 mm), height (50-100 % of insole thickness), and stiffness (0.25-2.25 MPa) | CT | Unknown | Walking (toe off) | Linearly elastic (bone, cartilage, flexor tendon, fascia), nonlinearly elastic (soft tissue, insole: #2 Plastizote, plug: Poron, sole: rubber) | Mechanical loading (the compressive load applied at the tibia, the moment simulating achilles tendon, flexor tendon prestress to assume the values of FDLF) | Frictionless contact (foot & insole, sole & ground), connect (insole & sole) | 2D, static | Plantar pressure | Plantar pressure | Pantar pressure |
| Hsu et al., 2008 | Conforming insole: conforming index (0, 3, 6, 15, 22, 33, 40, 54%) | CT | 4-node Tet (bone, skin, cartilage, soft tissue, insole, ground), link (ligament, fascia) | Standing | Linearly elastic (bone, cartilage, soft tissue, ligament, fascia, ground, insole: TPI-EVA) | Mechanical loading (GRF: 387 N, ATF: 193 N) | Constraint (the upper surfaces of tibia, fibula and soft tissue) | 3D, static | Plantar pressure, fascia stress, fascia strain | Plantar pressure, plantar contact area | Unknown |
| Goske et al., 2006 | Conforming insole: conforming index (flat, half conforming, full conforming), insole thickness (6.3, 9.5, 12.7 mm), insole materials (Poron Cushioning, Microcel Puff Lite, Microcel Puff) | MRI, scanning | Quad (heel pad) | Walking (early support) | Rigid (bone, ground), hyperelastic (heel pad, insole: Microcel Puff, Microcel Puff Lite, Poron Cushioning, midsole: Crepe), linearly elastic (upper: leather) | Mechanical loading (vertical load applied on the heel: 678 N) | Binding contact (upper & midsole, insole & the inside of shoe), frictional contact (heel pad & insole: 0.5, midsole & ground: 0.5) | 2D, static | Plantar pressure | Plantar pressure | Unknown |
| Cheung et al., 2005 | Conforming insole: conforming index (flat, full conforming), insole stiffness (0.3, 1.0, 1000 MPa) | MRI, modeling | Tet (bone, soft tissue, cartilage), truss (fascia, ligament), Hex (insole) | Standing | Linearly elastic (bone, soft tissue, cartilage, fascia, ligament), unknown (insole: PU, high density EVA, PP), rigid (ground) | Mechanical loading (vertical load applied on the insole: 350 N, ATF: 175 N) | Constraint (the upper surfaces of tibia, fibula, and soft tissue, the loading point at the foot COP: vertical movement only), frictional contact (foot & insole: 0.6), frictionaless contact (joint surface & joint surface) | 3D, static | Plantar pressure, plantar contact area, bone stress, fascia strain | Plantar pressure | Unknown |
| Chen et al., 2003 | Conforming insole: conforming index (full), insole-1, upper layer (PPT), middle layer (Microcel Puff), lower layer (Thermocork), insole-2, upper layer (medium Plastazote), lower layer (PPT) | CT | 4-node Tet (bone, soft tissue, cartilage), 2-node cable (ligament, fascia), 6-node wedge (insole) | Walking (mid-stance) | Linearly elastic (bone, soft tissue, cartilage, ligament), nonlinear elastic (insole: Microcel puff, PPT, thermocork, Medium plastazote), rigid (ground) | Displacement loading (ground: displacement 2.4 mm) | Constraint (the proximal surface of tibia, the front and back edges of insole), frictional contact (foot & insole: 0.3, insole & ground: 0.3) | 3D, quasi-  static | Plantar stress | Unknown | Unknown |
| CT, Computed Tomography; MRI, Magnetic Resonance Imaging; DM, Diabetes Mellitus; GRF, Ground Reaction Force; ATF, Achilles Tendon Force; BWF, Body Weight Force; JM, Joint Moment; JRF, Joint Reaction Force; NAJF, Net Ankle Joint Force; TAF, Tibialis Anterior Force; TPF, Tibialis Posterior Force; PBF, Peroneus Brevis Force; PLF, Peroneus Longus Force; FHLF, Flexor Hallucis Longus Force; FDLF, Flexor Digitorum Longus Force; PMGF, Peroneal Muscle Group Force; GSF, Gastrocnemius Soleus Force; SOLF, Soleus Force; GASF, Gastrocnemius Force; HAMF, Hamstring Force; QUADSF, Quadriceps Force; LRF, Lateral Retinaculum Force; MRF, Medial Retinaculum Force; COP, Center Of Pressure; GMAF, Gluteus Medius Abductor Force; Tet, Tetrahedral element; Hex, Hexahedral element; Quad, Quadrilateral Element; Tri, Triangle Element; PPT, Polypropylene Terephthalate; TPU, Thermoplastic Polyurethane; EVA, Ethylene Vinyl Acetate; TPC, Thermoplastic Copolyester; PE, Polyethylene; PVC, Polyvinyl Chloride; PU, Polyurethane; TPI, Trans-polyisoprene; PP, Polypropylene; | | | | | | | | | | | |
