# Supplementary material for: Finite element analysis of foot-insole biomechanics for insole design: a systematic review
Source: Front Bioeng Biotechnol. 2026 Oct 28;14:1994273. doi: 10.3389/fbioe.2026.1994273 (PMC13750745; doi:10.3389/fbioe.2026.1994273)
Supplement: Supplementary file 2 [file Supplementaryfile2.docx]

| **Appendix 2 Cross-Study Comparability Assessment** | | | | | | | | | | | | |
| --- | --- | --- | --- | --- | --- | --- | --- | --- | --- | --- | --- | --- |
| **Intervention** | **Outcome indicator** | **Authors** | **Model** | **Material properties^1^** | **Control group** | **Loading conditions** | **Participants^2^** | **Health status** | **Analysis type^3^** | **Model Dimensions** | **Movement** | **Comparability assessment** |
| Single-hole-structured insole | Soft tissue stress (including the heel pad) | Shaulian et al., 2021 | Bone, achilles tendon, skin, soft tissue, insole: EVA, cartilage | Linearly elastic (bone, achilles tendon), visco-hyperelastic (skin, soft tissue), hyperelastic (insole: EVA), unknown (cartilage) | Flat, standard insoles | Displacement loading (insole: upward displacement 7-10 mm to assume 320 N GRF) | Male | Diabetic patient with neuropathy | static | 3D | Walking (heel strike) | Comparable |
|  |  | Luo et al., 2011 | Skin, heel pad, insole, ground, bone | Hyperelastic (skin, heel pad, insole), linearly elastic (ground), rigid (bone) | Flat, standard insoles | Mechanical loading (BWF: 200 N) | Male | Healthy | Static | 2D | Standing | Not directly comparable |
|  |  | Shaulian et al., 2022 | Bone, cartilage, achilles tendon, skin, soft issue, insole & plug & insole cover & graded material: EVA | Linearly elastic (bone, cartilage, achilles tendon), visco-hyperelastic (skin, soft issue), hyperelastic (insole & plug & insole cover & graded material: EVA) | Flat, standard insoles | Displacement loading (insole: vertical displacement 7-10 mm to assume 320N GRF) | Male | Diabetic patient with neuropathy | Quasi-static | 3D | Walking (heel strike) | Comparable |
| Single-hole-structured insole | Plantar stress | Chanda et al., 2018 | DM foot, insole | Linearly elastic (DM foot), hyperelastic (insole) | No insoles (barefoot) | Contact loading (plantar pressure: 0.3 MPa) | Unknown | Diabetic patients with ulcers | Static | 3D | Standing | Comparable |
|  |  | Mala et al., 2025 | DM foot, insole: EVA, PU | Linearly elastic (DM foot), unknown (insole: EVA, PU) | No insoles (barefoot) | Contact loading (load applied on the top surface of the foot: 0.3 MPa) | Unknown | Diabetic patients with ulcers | Static | 3D | Standing | Comparable |
| Single-plug-structured insole | Plantar pressure | Geiger et al., 2023 | Soft tissue, bone, insole: TPC, sole, plug | Hyperelastic (soft tissue), linearly elastic (bone, insole: TPC, sole), unknown (plug) | No control group | Displacement loading (talus: vertical displacement 10 mm, with constraint on translation and rotation), mechanical loading (JRF and JM, with constraint on x-axis rotation) | Unknown | Healthy | Quasi-static | 3D | Walking (the early and late middle stance) | Not directly comparable |
|  |  | Actis et al., 2008 | Bone, cartilage, flexor tendon, fascia, soft tissue, insole: #2 Plastizote, plug: Poron, sole: rubber | Linearly elastic (bone, cartilage, flexor tendon, fascia), nonlinearly elastic (soft tissue, insole: #2 Plastizote, plug: Poron, sole: rubber) | Conforming insoles Without a Plug Structure | Mechanical loading (the compressive load applied at the tibia, the moment simulating achilles tendon, flexor tendon prestress to assume the values of FDLF) | Male | Diabetic patient with neuropathy | Static | 2D | Walking (toe off) | Not directly comparable |
| Single-hole-structured insole+multi-parameter variations (insole material) | Plantar stress | Chanda et al., 2018 | DM foot, insole | Linearly elastic (DM foot), hyperelastic (insole) | No insoles (barefoot) | Contact loading (plantar pressure: 0.3 MPa) | Unknown | Diabetic patients with ulcers | Static | 3D | Standing | Comparable |
|  |  | Mala et al., 2025 | DM foot, insole: EVA, PU | Linearly elastic (DM foot), unknown (insole: EVA, PU) | No insoles (barefoot) | Contact loading (load applied on the top surface of the foot: 0.3 MPa) | Unknown | Diabetic patients with ulcers | Static | 3D | Standing | Comparable |
| Variable stiffness insole | Plantar pressure | Wang et al., 2023 | Cartilage, fascia, bone, ground, healthy soft tissue, DM soft tissue, test insole: EVA | Linearly elastic (cartilage, fascia, bone, ground), hyperelastic (healthy soft tissue, DM soft tissue, test insole: EVA), unknown (ligament, insole: TPU) | No insoles (barefoot), flat standard insoles | Mechanical loading (GRF: 775 N, 680 N, 775 N, ATF: 1300 N, 532 N, 76 N) | Unknown | Diabetic patient with neuropathy | Static | 3D | Walking (heel strike, mid-stance, toe off) | Not directly comparable |
|  |  | Hu et al., 2023 | Bone, cartilage, fascia, ligament, insole: TPU, ground, soft tissue, DM soft tissue | Linearly elastic (bone, cartilage, fascia, ligament, insole: TPU, ground), hyperelastic (soft tissue, DM soft tissue) | Flat, standard insoles | Mechanical loading (GRF: 380 N, ATF: 285 N) | Male | Healthy | Static | 3D | Standing | Not directly comparable |
|  |  | Zhu et al., 2021 | Bone, soft tissue, DM soft tissue, test insole, insole: TPU, ground | Linearly elastic (bone, soft tissue, DM soft tissue), unknown (test insole, insole: TPU, ground) | Flat, standard insoles | Mechanical loading (GRF, ATF) | Male/female | Diabetic patient with neuropathy | Static | 3D | Standing | Comparable |
|  |  | Jafarzadeh et al., 2021 | Bone, ligament, fascia, ground, insole: initial & EVA, soft tissue | Linearly elastic (bone, ligament, fascia, ground, insole: initial & EVA), hyperelastic (soft tissue) | Flat, standard insoles | Mechanical loading (GRF: 395 N, ATF) | Male | Diabetic patient with neuropathy | Static | 3D | Standing | Comparable |
|  |  | Tang et al., 2019 | Bone, soft tissue, insole: TPU, ground | Linearly elastic (bone, soft tissue), unknown (insole: TPU), rigid (ground) | Flat, standard insoles | Mechanical loading (GRF, ATF) | Unknown | Healthy | Static | 3D | Standing | Not directly comparable |
| Variable stiffness insole | Soft tissue stress | Wang et al., 2023 | Cartilage, fascia, bone, ground, healthy soft tissue, DM soft tissue, test insole: EVA | Linearly elastic (cartilage, fascia, bone, ground), hyperelastic (healthy soft tissue, DM soft tissue, test insole: EVA), unknown (ligament, insole: TPU) | No insoles (barefoot), flat standard insoles | Mechanical loading (GRF: 775 N, 680 N, 775 N, ATF: 1300 N, 532 N, 76 N) | Unknown | Diabetic patient with neuropathy | Static | 3D | Walking (heel strike, mid-stance, toe off) | Not directly comparable |
|  |  | Hu et al., 2023 | Bone, cartilage, fascia, ligament, insole: TPU, ground, soft tissue, DM soft tissue | Linearly elastic (bone, cartilage, fascia, ligament, insole: TPU, ground), hyperelastic (soft tissue, DM soft tissue) | Flat, standard insoles | Mechanical loading (GRF: 380 N, ATF: 285 N) | Male | Healthy | Static | 3D | Standing | Not directly comparable |
|  |  | Zhu et al., 2021 | Bone, soft tissue, DM soft tissue, test insole, insole: TPU, ground | Linearly elastic (bone, soft tissue, DM soft tissue), unknown (test insole, insole: TPU, ground) | Flat, standard insoles | Mechanical loading (GRF, ATF) | Male/female | Diabetic patient with neuropathy | Static | 3D | Standing | Not directly comparable |
| Variable stiffness insole+Conforming insole | Plantar pressure | Tang et al., 2019 | Bone, soft tissue, insole: TPU, ground | Linearly elastic (bone, soft tissue), unknown (insole: TPU), rigid (ground) | Flat, standard insoles | Mechanical loading (GRF, ATF) | Unknown | Healthy | Static | 3D | Standing | Not directly comparable |
|  |  | Cracknell et al., 2025 | Bone, soft tissue, insole: porous structure, insole: fluid | Linearly elastic (bone), hyperelastic (soft tissue, insole: porous structure), rigid (ground), unknown (insole: fluid) | Conforming insoles with or without internal fluid filling | Mechanical loading (reaction force and moment) | Unknown | Unknown | Static | 3D | Walking (heel strike, foot flat, mid-stance, terminal stance, heel off, toe off) | Not directly comparable |
| Arch support insole | Plantar pressure | Cheung et al., 2008 | Bone, ligament, fascia, cartilage, soft tissue, insole & midsole & outsole: PU & EVA, ground | Linearly elastic (bone, ligament, fascia, cartilage), hyperelastic (soft tissue, insole & midsole & outsole: PU & EVA), unknown (ground) | Flat, standard insoles | Mechanical loading (GRF: 550 N, ATF: 700 N, muscle force: TPF: 70 N, FHLF: 40 N, FDLF: 30 N, PBF: 30 N, PLF: 40 N, LRF: 50 N, MRF: 50 N) | Male | Healthy | Static | 3D | Walking (mid-stance) | Not directly comparable |
|  |  | Peng et al., 2021 | Ground, bone, soft tissue, insole, midsole, fascia, skin, ligament, muscle | Hex (ground), 4-node Tet (bone, soft tissue, insole, midsole, fascia), 3-node Tri membrane (skin), truss (ligament), connector (muscle) | No control group | Mechanical loading (GRF, ATF, muscle force: TAF, TPF, PBF, PLF, FHLF, FDLF) | Male | Patient with flat feet | Static | 3D | Walking (the first peak of vertical GRF, GRF valley, the second peak of vertical GRF) | Not directly comparable |
|  |  | Su et al., 2017 | Bone, soft tissue, fascia, cartilage, ligament, skin, ground | Linearly elastic (bone, soft tissue, fascia, cartilage, ligament, skin), unknown (ground) | No insoles (barefoot) | Mechanical loading (ATF: 375 N), displacement loading (navicular bone displacement: vertical displacement) | Adolescent male | Patient with flat feet | Static | 3D | Standing | Not directly comparable |
|  |  | Alsaleh et al., 2025 | Insole: EVA, TPU, rubber, ground, foot | Linearly elastic (insole: EVA, TPU, rubber), rigid (ground), unknown (foot) | No insoles (barefoot) | Mechanical loading (GRF: 421.8N) | Adolescent female | Patient with hereditary spastic paraplegia, accompanied by clubfoot, a knee-bending gait, and hallux valgus | Static | 3D | Walking (mid-stance) | Not directly comparable |
| Arch support insole | Ligament stress and arch height | Peng et al., 2021 | Ground, bone, soft tissue, insole, midsole, fascia, skin, ligament, muscle | Hex (ground), 4-node Tet (bone, soft tissue, insole, midsole, fascia), 3-node Tri membrane (skin), truss (ligament), connector (muscle) | No control group | Mechanical loading (GRF, ATF, muscle force: TAF, TPF, PBF, PLF, FHLF, FDLF) | Male | Patient with flat feet | Static | 3D | Walking (the first peak of vertical GRF, GRF valley, the second peak of vertical GRF) | Not directly comparable |
|  |  | Su et al., 2017 | Bone, soft tissue, fascia, cartilage, ligament, skin, ground | Linearly elastic (bone, soft tissue, fascia, cartilage, ligament, skin), unknown (ground) | No insoles (barefoot) | Mechanical loading (ATF: 375 N), displacement loading (navicular bone displacement: vertical displacement) | Adolescent male | Patient with flat feet | Quasi-static | 3D | Standing | Not directly comparable |
| Arch support insole+multi-parameter variations | Plantar pressure | Cheung et al., 2008 | Bone, ligament, fascia, cartilage, soft tissue, insole & midsole & outsole: PU & EVA, ground | Linearly elastic (bone, ligament, fascia, cartilage), hyperelastic (soft tissue, insole & midsole & outsole: PU & EVA), unknown (ground) | Flat, standard insoles | Mechanical loading (GRF: 550 N, ATF: 700 N, muscle force: TPF: 70 N, FHLF: 40 N, FDLF: 30 N, PBF: 30 N, PLF: 40 N, LRF: 50 N, MRF: 50 N) | Male | Healthy | Static | 3D | Walking (mid-stance) | Not directly comparable |
|  |  | Su et al., 2017 | Bone, soft tissue, fascia, cartilage, ligament, skin, ground | Linearly elastic (bone, soft tissue, fascia, cartilage, ligament, skin), unknown (ground) | No insoles (barefoot) | Mechanical loading (ATF: 375 N), displacement loading (navicular bone displacement: vertical displacement) | Adolescent male | Patient with flat feet | Quasi-static | 3D | Standing | Not directly comparable |
|  |  | Alsaleh et al., 2025 | Insole: EVA, TPU, rubber, ground, foot | Linearly elastic (insole: EVA, TPU, rubber), rigid (ground), unknown (foot) | No insoles (barefoot) | Mechanical loading (GRF: 421.8N) | Adolescent female | Patient with hereditary spastic paraplegia, accompanied by clubfoot, a knee-bending gait, and hallux valgus | Static | 3D | Walking (mid-stance) | Not directly comparable |
| Conforming insole | Plantar stress | Serrato-Pedrosa et al., 2024a | Skin, muscle, ground, insole: TPU | Linearly elastic (skin, muscle), unknown (ground, insole: TPU) | No insoles (barefoot) | Displacement loading (ground: vertical displacement 5 mm to assume 400 N GRF) | Male | Healthy | Static | 3D | Standing | Not directly comparable |
|  |  | Serrato-Pedrosa et al., 2024b | Skin, muscle, insole: TPU, ground | Linearly elastic (skin, muscle, insole: TPU), rigid (ground) | No insoles (barefoot) | Displacement loading (ground: vertical displacement 6, 7, 5.5, 8, 8.5 mm to assume GRF) | Male | Healthy | Quasi-static | 3D | Walking (heel strike, loading response, mid-stance, heel rise, toe off) | Not directly comparable |
|  |  | Sarikhani et al., 2016 | Bone, insole, soft tissue | Linearly elastic (bone, insole), hyperelastic (soft tissue) | Flat, standard insoles | Mechanical loading (load applied on the achilles tendon) | Unknown | Unknown | Unknown | 3D | Unknown | Not directly comparable |
|  |  | Chen et al., 2003 | Bone, soft tissue, cartilage, ligament, insole: Microcel puff, PPT, thermocork, Medium plastazote, ground | Linearly elastic (bone, soft tissue, cartilage, ligament), nonlinear elastic (insole: Microcel puff, PPT, thermocork, Medium plastazote), rigid (ground) | Flat, standard insoles | Displacement loading (ground: displacement 2.4 mm) | Male | Unknown | Quasi-static | 3D | Walking (mid-stance) | Not directly comparable |
| Conforming insole | Plantar pressure | Zhang et al., 2017 | Bone, soft tissue, fascia, ligament, gastrocnemius, meniscus, ground, insole: PU & EVA | Linearly elastic (bone, soft tissue, fascia, ligament, gastrocnemius, meniscus, ground, insole: PU & EVA) | No insoles (barefoot), flat standard insoles | Mechanical loading (upward load applied on the ground: 320 N, muscle force: gastrocnemius force: 240 N) | Female | Patient with heel pain | Static | 3D | Standing | Not directly comparable |
|  |  | Cheung et al., 2005 | Bone, soft tissue, cartilage, fascia, ligament, insole: PU, high density EVA, PP, ground | Linearly elastic (bone, soft tissue, cartilage, fascia, ligament), unknown (insole: PU, high density EVA, PP), rigid (ground) | Flat, standard insoles | Mechanical loading (vertical load applied on the insole: 350 N, ATF: 175 N) | Male | Healthy | Static | 3D | Standing | Comparable |
|  |  | Hsu et al., 2008 | Bone, cartilage, soft tissue, ligament, fascia, ground, insole: TPI-EVA | Linearly elastic (bone, cartilage, soft tissue, ligament, fascia, ground, insole: TPI-EVA) | Flat, standard insoles | Mechanical loading (GRF: 387 N, ATF: 193 N) | Male | Healthy | Static | 3D | Standing | Comparable |
|  |  | Goske et al., 2008 | Bone, ground, heel pad, insole: Microcel Puff, Microcel Puff Lite, Poron Cushioning, midsole: Crepe, upper: leather | Rigid (bone, ground), hyperelastic (heel pad, insole: Microcel Puff, Microcel Puff Lite, Poron Cushioning, midsole: Crepe), linearly elastic (upper: leather) | No insoles (barefoot) | Mechanical loading (vertical load applied on the heel: 678 N) | Male | Healthy | Static | 2D | Walking (early support) | Not directly comparable |
| Conforming insole+multi-parameter variations (insole thickness and material hardness) | Plantar pressure | Zhang et al., 2017 | Bone, soft tissue, fascia, ligament, gastrocnemius, meniscus, ground, insole: PU & EVA | Linearly elastic (bone, soft tissue, fascia, ligament, gastrocnemius, meniscus, ground, insole: PU & EVA) | No insoles (barefoot), flat standard insoles | Mechanical loading (upward load applied on the ground: 320 N, muscle force: gastrocnemius force: 240 N) | Female | Patient with heel pain | Static | 3D | Standing | Not directly comparable |
|  |  | Cheung et al., 2005 | Bone, soft tissue, cartilage, fascia, ligament, insole: PU, high density EVA, PP, ground | Linearly elastic (bone, soft tissue, cartilage, fascia, ligament), unknown (insole: PU, high density EVA, PP), rigid (ground) | Flat, standard insoles | Mechanical loading (vertical load applied on the insole: 350 N, ATF: 175 N) | Male | Healthy | Static | 3D | Standing | Not directly comparable |
|  |  | Goske et al., 2008 | Bone, ground, heel pad, insole: Microcel Puff, Microcel Puff Lite, Poron Cushioning, midsole: Crepe, upper: leather | Rigid (bone, ground), hyperelastic (heel pad, insole: Microcel Puff, Microcel Puff Lite, Poron Cushioning, midsole: Crepe), linearly elastic (upper: leather) | No insoles (barefoot) | Mechanical loading (vertical load applied on the heel: 678 N) | Male | Healthy | Static | 2D | Walking (early support) | Not directly comparable |
| 1.Linear elastic and nonlinear soft-tissue material models were considered not directly comparable. For studies involving other soft tissues, such as the Achilles tendon, ligaments, or fascia, the material models were also required to be consistent.  2:Males and females are considered comparable, while adolescents and middle-aged individuals are considered non-comparable.  3:Static and quasi-static loading are considered fundamentally comparable. | | | | | | | | | | | | |
