# Supplementary material for: Finite element analysis of foot-insole biomechanics for insole design: a systematic review
Source: Front Bioeng Biotechnol. 2026 Oct 28;14:1994273. doi: 10.3389/fbioe.2026.1994273 (PMC13750745; doi:10.3389/fbioe.2026.1994273)
Supplement: Supplementary file 3 [file Supplementaryfile3.docx]

| **Appendix 3 Effect of interventions on outcome indicator** | | | | |
| --- | --- | --- | --- | --- |
| **Intervention** | **Outcome indicator** | **High quality*** | **Low quality*** | **Best evidence synthesis** |
| Non-featured insole: an insole with a metatarsal pad, a toe pad, and arch support | Fascia stress | + ^Wang et al., 2025^ |  | Limited |
|  | Bone stress | + ^Wang et al., 2025^ |  | Limited |
| Non-featured insole | Plantar stress | ± ^Lin et al., 2025^ |  | Limited |
|  | Foot deformation | ± ^Lin et al., 2025^ |  | Limited |
|  | Metatarsal stress | ± ^Lin et al., 2025^ |  | Limited |
|  | Bone tuberosity stress | ± ^Lin et al., 2025^ |  | Limited |
| Non-featured insole: an insole with layered modular | Plantar pressure |  | + ^Niu et al., 2020^ | Limited |
| Non-featured insole: an insole with pressurized bubbles | Plantar contact pressure |  | + ^Strayer et al., 2020^ | Limited |
| Non-featured insole: Yonex Active Pro Truactive, Victor VT-XD 8, Li-Ning L6200LA | Insole stress | + ^Ab Rashid et al., 2022^ |  | Limited |
|  | Bone stress | + ^Ab Rashid et al., 2022^ |  | Limited |
|  | Bone displacement | ? ^Ab Rashid et al., 2022^ |  | Limited |
|  | Bone rotation angle | ? ^Ab Rashid et al., 2022^ |  | Limited |
| Non-featured insole | Plantar pressure | + ^Yang et al., 2022^ |  | Limited |
| Non-featured insole | Plantar pressure | + ^Nouman et al., 2021^ |  | Limited |
|  | Plantar frictional stress | + ^Nouman et al., 2021^ |  | Limited |
| Non-featured insole | Plantar pressure |  | + ^Nouman et al., 2023^ | Limited |
|  | Plantar frictional stress |  | + ^Nouman et al., 2023^ | Limited |
| Multi-hole-structured insole | Plantar pressure | + ^Cao et al., 2021^ |  | Limited |
|  | Soft tissue stress | + ^Cao et al., 2021^ |  | Limited |
| Single-hole-structured insole | Soft tissue stress (including the heel pad) | + ^Shaulian et al., 2021^ | + ^Shaulian et al., 2022^ | Moderate |
|  |  |  | + ^Luo et al., 2011^ |  |
|  | Total stress concentration exposure of soft tissue | + ^Shaulian et al., 2021^ |  | Limited |
|  | Plantar stress | + ^Chanda et al., 2018^ | + ^Mala et al., 2025^ | Moderate |
|  | Heel pad strain |  | + ^Luo et al., 2011^ | Limited |
|  | Strain energy density of heel pad |  | + ^Luo et al., 2011^ | Limited |
| Multi-plug-structured insole | Plantar pressure |  | + ^Actis et al., 2008^ | Limited |
| Single-plug-structured insole | Plantar pressure | + ^Geiger et al., 2023^ |  | Limited |
|  |  |  | + ^Actis et al., 2008^ |  |
|  | Soft tissue stress |  | + ^Shaulian et al., 2022^ | Limited |
|  | Total stress concentration exposure of soft tissue |  | + ^Shaulian et al., 2022^ | Limited |
| Hole/plug-structured-insole+novel graded stiffness | Soft tissue stress |  | + ^Shaulian et al., 2022^ | Limited |
|  | Total stress concentration exposure of soft tissue |  | + ^Shaulian et al., 2022^ | Limited |
| Single-hole-structured insole+multi-parameter variations (insole material) | Plantar stress | + ^Chanda et al., 2018^ | + ^Mala et al., 2025^ | Moderate |
| Variable stiffness insole | Plantar pressure |  | + ^Zhu et al., 2021; Jafarzadeh et al., 2021^ | Moderate |
|  |  | + ^Tang et al., 2019^ |  |  |
|  |  |  | + ^Wang et al., 2023^ |  |
|  |  |  | + ^Hu et al., 2023^ |  |
|  | Soft tissue stress |  | + ^Wang et al., 2023^ | Limited |
|  |  |  | + ^Hu et al., 2023^ |  |
|  |  |  | + ^Zhu et al., 2021^ |  |
|  | Bone stress |  | + ^Hu et al., 2023^ | Limited |
|  | Cartilage stress |  | + ^Wang et al., 2023^ | Limited |
|  | Total stress concentration exposure of soft tissue |  | + ^Zhang et al., 2025^ | Limited |
|  | Compressive stress  of heel pad |  | + ^Zhang et al., 2025^ | Limited |
|  | Tensile stress of skin |  | + ^Zhang et al., 2025^ | Limited |
| Variable stiffness insole+Conforming insole | Plantar pressure | + ^Tang et al., 2019^ |  | Limited |
|  |  | + ^Cracknell et al., 2025^ |  |  |
|  | Plantar contact area | + ^Tang et al., 2019^ |  | Limited |
| Variable stiffness/thickness insole+Conforming insole | Plantar pressure | + ^Li et al., 2024^ |  | Limited |
| Variable stiffness/shape insole | Plantar pressure |  | + ^Jafarzadeh et al., 2021^ | Limited |
| Lateral wedge insole | Subtalar joint contact area |  | ± ^Zhou et al., 2017^ | Limited |
|  | Subtalar joint stress |  | ± ^Zhou et al., 2017^ | Limited |
|  | Plantar pressure |  | ± ^Du et al., 2024^ | Limited |
|  | Joint contact pressure |  | ± ^Du et al., 2024^ | Limited |
|  | Bone stress |  | ± ^Du et al., 2024^ | Limited |
|  | Ligament stress |  | ± ^Du et al., 2024^ | Limited |
|  | Cartilage stress | + ^Liu et al., 2013^ |  | Limited |
|  | Meniscus stress | + ^Liu et al., 2013^ |  | Limited |
|  | Meniscus contact force | ± ^Liu et al., 2013^ |  | Limited |
| Medial wedge insole | Plantar pressure | ± ^Guo et al., 2016^ |  | Limited |
|  | Bone stress | ± ^Guo et al., 2016^ |  | Limited |
|  | Ligament tension | ± ^Guo et al., 2016^ |  | Limited |
|  | Fascia stress | ± ^Guo et al., 2016^ |  | Limited |
| Arch support insole | Plantar pressure | ± ^Cheung et al., 2008^ |  | Limited |
|  |  | ± ^Peng et al., 2021^ |  |  |
|  |  | ± ^Su et al., 2017^ |  |  |
|  |  |  | ± ^Alsaleh et al., 2025^ |  |
|  | Fascia strain | ± ^Peng et al., 2021^ |  | Limited |
|  | Ligament stress | ± ^Su et al., 2017^ |  | Limited |
|  |  | ± ^Su et al., 2017^ |  |  |
|  | Arch height | + ^Peng et al., 2021^ |  | Limited |
|  |  | ± ^Su et al., 2017^ |  |  |
|  | Cartilage stress | ± ^Su et al., 2017^ |  | Limited |
|  | Plantar stress |  | ± ^Alsaleh et al., 2025^ | Limited |
|  | Plantar strain |  | ± ^Alsaleh et al., 2025^ | Limited |
|  | Plantar deformation |  | ± ^Alsaleh et al., 2025^ | Limited |
| Arch support insole+multi-parameter variations | Arch height | + ^Su et al., 2017^ |  | Limited |
|  | Plantar pressure | ± ^Cheung et al., 2008^ |  | Limited |
|  |  | ± ^Su et al., 2017^ |  |  |
|  |  |  | ± ^Alsaleh et al., 2025^ |  |
|  | Cartilage stress | ± ^Su et al., 2017^ |  | Limited |
|  | Ligament stress | + ^Su et al., 2017^ |  | Limited |
|  | Plantar stress |  | ± ^Alsaleh et al., 2025^ | Limited |
|  | Plantar strain |  | ± ^Alsaleh et al., 2025^ | Limited |
|  | Plantar deformation |  | ± ^Alsaleh et al., 2025^ | Limited |
| Conforming insole | Plantar stress | + ^Serrato-Pedrosa et al., 2024a^ |  | Limited |
|  |  | + ^Serrato-Pedrosa et al., 2024b^ |  |  |
|  |  |  | + ^Sarikhani et al., 2016^ |  |
|  |  |  | + ^Chen et al., 2003^ |  |
|  | Plantar deformation | + ^Serrato-Pedrosa et al., 2024b^ |  | Limited |
|  | Insole stress | + ^Serrato-Pedrosa et al., 2024a^ |  | Limited |
|  | Metatarsal stress | + ^Zhang et al., 2017^ |  | Limited |
|  | Plantar pressure | + ^Cheung et al., 2005^ | + ^Hsu et al., 2008^ | Moderate |
|  |  | + ^Zhang et al., 2017^ |  |  |
|  |  | + ^Goske et al., 2006^ |  |  |
|  | Fascia stress |  | + ^Hsu et al., 2008^ | Limited |
|  | Fascia strain |  | + ^Hsu et al., 2008^ | Limited |
| Conforming insole+multi-parameter variations (insole thickness and material hardness) | Plantar pressure | + ^Zhang et al., 2017^ |  | Limited |
|  |  | + ^Cheung et al., 2005^ |  |  |
|  |  | + ^Goske et al., 2006^ |  |  |
|  | Plantar contact area | + ^Cheung et al., 2005^ |  | Limited |
|  | Bone stress (the forefoot and midfoot area) | + ^Cheung et al., 2005^ |  | Limited |
|  | Fascia strain | + ^Cheung et al., 2005^ |  | Limited |
|  | Metatarsal stress | + ^Zhang et al., 2017^ |  | Limited |
| Conforming insole+multi-parameter variations (insole thickness, material hardness, and heel’s height) | Plantar stress |  | + ^Sarikhani et al., 2016^ | Limited |
| Conforming insole+layered material design | Plantar stress |  | + ^Chen et al., 2003^ | Limited |
| Arch support insole+wedge insole+multi-parameter variations (insole stiffness) | Plantar stress (surface) | + ^Zhang et al., 2020b^ |  | Limited |
|  | Soft tissue stress | + ^Zhang et al., 2020b^ |  | Limited |
|  | Cartilage stress | + ^Zhang et al., 2020b^ |  | Limited |
|  | Fascia stress | + ^Zhang et al., 2020b^ |  | Limited |
| Arch support insole+wedge insole+multi-parameter variations (insole stiffness) | Cartilage stress | + ^Zhang et al., 2020a^ |  | Limited |
|  | Plantar pressure | + ^Zhang et al., 2020a^ |  | Limited |
|  | Bone stress | + ^Zhang et al., 2020a^ |  | Limited |
| Arch support insole+wedge insole+multi-parameter variations (insole stiffness and heel cup height) | Plantar pressure | + ^Peng et al., 2022^ |  | Limited |
|  | Fascia strain | + ^Peng et al., 2022^ |  | Limited |
| Partial insole (heel insole) | Plantar stress | + ^Zhang et al., 2018^ |  | Limited |
|  | Soft tissue stress | + ^Zhang et al., 2018^ |  | Limited |
|  | Fascia stress | + ^Zhang et al., 2018^ |  | Limited |
| Partial insole (metatarsal insole) | Plantar pressure | ± ^Chen et al., 2015^ |  | Limited |
|  | Soft tissue strain | ± ^Chen et al., 2015^ |  | Limited |
|  | Bone stress | − ^Chen et al., 2015^ |  | Limited |
|  | Fascia tensile stress | ± ^Chen et al., 2015^ |  | Limited |
|  | Toe pad compressive strain | − ^Chen et al., 2015^ |  | Limited |
| *High quality studies are studies with MQSSFE score ≥ 22.2; low quality studies are studies with MQSSFE score < 22.2.  +, positive effect of the intervention on the outcome measure;  −, negative effect of the intervention on the outcome measure;  =, no effect of the intervention on the outcome measure;  ±, mixed or inconsistent effects, indicating that both positive and negative outcomes were reported depending on intervention parameters.  ? , unclear or indeterminate effect, used when the biomechanical or clinical implication of the outcome measure cannot be confidently classified as positive, negative, or mixed. | | | | |
