# Supplementary material for: Finite element analysis of foot-insole biomechanics for insole design: a systematic review
Source: Front Bioeng Biotechnol. 2026 Oct 28;14:1994273. doi: 10.3389/fbioe.2026.1994273 (PMC13750745; doi:10.3389/fbioe.2026.1994273)
Supplement: Supplementary file 4 [file Supplementaryfile4.docx]

| **Appendix 4. Methodological quality assessment for the included studies.** | | | | | | | | | | | |
| --- | --- | --- | --- | --- | --- | --- | --- | --- | --- | --- | --- |
|  | Alsaleh et al., 2025 | Cracknell et al., 2025 | Lin et al., 2025 | Mala et al., 2025 | Wang et al., 2025 | Zhang et al., 2025 | Serrato-Pedrosa et al., 2024a | Serrato-Pedrosa et al., 2024b | Li et al., 2024 | Du et al., 2024 | Geiger et al., 2023 |
| Item 1 | + | + | + | + | + | + | + | + | + | + | + |
| Item 2 | + | + | + | - | + | + | + | + | + | + | + |
| Item 3 | + | + | + | - | + | + | + | + | + | + | + |
| Item 4 | + | + | + | + | + | + | + | - | + | + | - |
| Item 5 | + | + | + | - | + | + | + | + | + | + | + |
| Item 6 | + | + | + | + | + | + | + | + | + | + | + |
| Item 7 | + | + | + | + | + | + | + | + | + | + | + |
| Item 8 | - | + | - | - | + | + | - | - | + | + | + |
| Item 9 | + | - | + | - | + | - | + | + | + | + | + |
| Item10 | + | - | + | - | + | - | + | + | + | + | + |
| Item11 | + | - | - | - | - | - | - | - | - | - | - |
| Item12 | + | + | + | + | + | + | + | + | + | + | + |
| Item13 | + | + | - | + | + | - | + | + | + | - | + |
| Item14 | - | - | + | - | + | - | + | - | - | - | + |
| Item15 | - | + | - | - | + | - | - | - | - | - | + |
| Item16 | + | + | + | - | + | - | + | + | + | - | + |
| Item17 | + | + | + | + | + | + | - | + | + | + | + |
| Item18 | - | - | + | - | + | - | + | + | - | - | + |
| Item19 | + | + | - | + | + | + | + | + | + | + | + |
| Item20 | - | + | + | - | + | + | + | + | - | + | + |
| Item21 | + | + | + | - | + | - | + | + | + | - | + |
| Item22 | - | + | + | - | + | - | + | + | - | + | + |
| Item23 | + | + | + | - | + | - | - | + | - | + | + |
| Item24 | - | - | - | - | - | - | - | - | - | - | - |
| Item25 | + | + | + | - | + | - | - | + | - | + | + |
| Item26 | - | + | - | + | - | - | + | + | - | - | + |
| Item27 | + | - | + | + | - | + | + | + | + | - | + |
| Item28 | - | + | + | + | + | + | + | + | + | + | + |
| Item29 | - | + | + | - | + | + | + | + | + | + | + |
| Item30 | - | - | + | + | + | + | + | + | + | + | + |
| Item31 | - | - | + | + | - | + | + | + | - | - | + |
| Item32 | - | + | - | + | - | - | - | + | + | - | + |
| Item33 | - | + | + | - | - | - | - | + | + | + | - |
| Item34 | - | + | - | - | - | - | - | + | - | - | + |
| Item35 | + | + | + | - | - | + | + | + | - | - | - |
| Item36 | + | + | - | - | + | - | + | - | - | - | + |
| Item37 | + | + | - | + | - | + | + | + | + | + | + |
| Sum | 22 | 28 | 26 | 15 | 27 | 19 | 27 | 30 | 23 | 22 | 32 |

| **Appendix 4. Methodological quality assessment for the included studies.** | | | | | | | | | | | |
| --- | --- | --- | --- | --- | --- | --- | --- | --- | --- | --- | --- |
| Nouman et al., 2023 | Wang et al., 2023 | Hu et al., 2023 | Shaulian et al., 2022 | AB Rashid et al., 2022 | Yang et al., 2022 | Peng et al., 2022 | Peng et al., 2021 | Jafarzadeh et al., 2021 | Zhu et al., 2021 | Cao et al., 2021 | Nouman et al., 2021 |
| + | + | + | + | + | + | + | + | + | + | + | + |
| + | + | - | + | + | + | + | + | + | + | + | + |
| + | + | + | + | + | + | + | + | + | - | + | + |
| - | + | + | + | + | + | + | + | + | - | + | - |
| + | + | + | + | + | + | + | + | + | - | + | + |
| + | + | + | + | + | + | + | + | + | + | + | + |
| + | + | + | + | + | + | + | + | + | + | + | + |
| + | + | - | + | + | + | + | + | - | - | - | - |
| - | - | + | - | + | + | + | + | + | + | + | - |
| + | - | - | - | + | + | + | + | + | - | + | + |
| - | + | - | - | + | + | + | + | + | - | - | - |
| + | + | + | + | + | + | + | + | + | + | + | - |
| + | - | - | + | + | + | + | + | + | - | + | + |
| - | + | + | - | + | + | - | + | + | - | + | - |
| + | - | - | - | - | + | + | + | + | - | + | - |
| + | + | - | - | + | + | + | + | + | - | - | + |
| + | + | - | + | + | + | + | + | + | - | + | + |
| - | - | + | + | + | + | - | + | - | - | - | + |
| + | + | + | + | + | + | + | + | + | + | + | + |
| + | - | + | - | + | + | - | - | + | + | + | + |
| + | + | + | + | + | + | + | - | + | + | + | + |
| - | + | - | - | + | + | + | + | - | - | - | - |
| - | + | - | - | - | + | + | + | - | - | - | - |
| - | - | - | - | - | + | + | + | - | - | - | - |
| - | + | - | - | - | + | + | + | - | - | - | - |
| - | - | - | + | + | + | + | + | - | - | + | - |
| + | - | - | + | + | + | - | + | - | - | - | + |
| - | + | + | - | + | + | + | + | - | + | + | + |
| - | + | + | - | - | + | - | + | - | + | + | + |
| - | + | + | - | + | + | + | + | - | - | + | + |
| - | - | - | + | + | - | + | + | - | + | - | - |
| + | - | - | + | + | + | + | + | - | + | - | + |
| + | - | + | + | - | + | - | + | + | - | + | + |
| - | - | - | - | - | - | - | + | - | + | - | + |
| + | - | - | + | + | + | + | + | + | + | - | + |
| - | - | - | + | + | + | + | - | + | + | - | - |
| + | + | + | + | + | + | + | + | - | + | + | + |
| 21 | 22 | 18 | 22 | 30 | 35 | 30 | 34 | 22 | 17 | 23 | 23 |

| **Appendix 4. Methodological quality assessment for the included studies.** | | | | | | | | | | | |
| --- | --- | --- | --- | --- | --- | --- | --- | --- | --- | --- | --- |
| Shaulian et al., 2021 | Zhang et al., 2020b | Zhang et al., 2020a | Niu et al., 2020 | Strayer et al., 2020 | Tang et al., 2019 | Zhang et al., 2018 | Chanda et al., 2018 | Zhang et al., 2017 | Zhou et al., 2017 | Su et al., 2017 | Guo et al., 2016 |
| + | + | + | - | + | + | + | + | + | + | + | + |
| + | + | + | - | + | + | + | + | + | + | + | + |
| + | + | + | + | + | + | + | + | + | + | + | + |
| + | + | + | - | - | + | + | + | + | + | + | + |
| + | + | + | + | + | + | + | + | + | + | - | - |
| + | + | + | + | + | + | + | + | + | + | + | + |
| + | + | + | + | + | + | + | + | + | + | + | + |
| + | + | + | - | - | + | + | - | + | - | - | - |
| - | + | + | + | - | - | + | - | + | + | + | + |
| - | + | + | + | - | - | + | - | + | + | + | - |
| - | + | + | - | - | - | + | - | + | - | - | - |
| + | + | + | + | - | + | + | + | + | + | + | + |
| + | + | + | - | - | + | + | + | + | - | + | + |
| + | + | + | + | - | - | + | + | + | + | + | - |
| + | + | + | + | - | - | + | + | + | + | + | - |
| - | - | + | - | - | + | + | + | - | - | + | + |
| + | + | - | + | + | + | + | - | + | + | - | + |
| + | - | - | - | + | - | - | + | - | + | + | - |
| + | + | + | + | + | + | - | + | + | + | + | + |
| - | + | + | + | + | + | + | + | + | - | - | + |
| + | + | + | + | + | + | + | + | + | + | + | + |
| - | + | + | - | - | - | - | - | - | + | - | - |
| - | - | - | - | - | + | - | - | - | - | + | - |
| - | - | + | - | - | - | - | - | + | - | - | - |
| - | - | - | - | - | + | - | - | - | - | + | - |
| + | + | - | - | - | - | - | + | - | - | + | - |
| - | - | - | - | + | - | - | + | - | - | + | + |
| + | + | + | - | + | + | + | + | + | + | + | + |
| - | + | + | - | - | + | + | - | + | - | + | + |
| + | + | + | - | + | + | + | + | + | + | + | + |
| + | - | - | - | + | + | + | + | - | + | - | - |
| + | - | + | + | - | - | - | + | - | - | - | + |
| + | - | + | + | - | + | - | - | - | - | + | + |
| - | - | - | - | - | - | - | - | - | - | - | + |
| + | - | + | + | - | - | - | - | - | - | + | - |
| - | - | + | - | + | + | - | + | - | - | - | + |
| + | + | + | + | + | + | + | + | + | + | + | + |
| 24 | 25 | 29 | 17 | 17 | 24 | 24 | 24 | 24 | 21 | 26 | 23 |

| **Appendix 4. Methodological quality assessment for the included studies.** | | | | | | | | | | |
| --- | --- | --- | --- | --- | --- | --- | --- | --- | --- | --- |
| Sarikhani et al., 2016 | Chen et al., 2015 | Liu et al., 2013 | Luo et al., 2011 | Cheung et al., 2008 | Actis et al., 2008 | Hsu et al., 2008 | Goske et al., 2006 | Cheung et al., 2005 | Chen et al., 2003 | Sum |
| + | + | + | + | + | + | + | + | + | + | 44 |
| + | + | + | + | + | + | + | + | + | + | 42 |
| + | + | + | + | + | + | + | + | + | + | 43 |
| + | + | + | + | + | + | - | + | + | + | 37 |
| - | + | + | + | + | + | + | + | + | + | 40 |
| + | + | + | + | + | + | + | + | + | + | 45 |
| + | + | + | + | + | + | + | + | + | + | 45 |
| - | + | - | + | + | - | + | - | + | + | 25 |
| - | - | + | - | + | - | - | + | + | - | 28 |
| - | - | + | - | - | - | + | + | + | - | 27 |
| - | - | - | - | - | - | - | - | - | - | 11 |
| + | + | + | + | + | + | + | + | + | + | 43 |
| + | - | + | - | - | - | - | - | - | - | 28 |
| - | - | + | - | + | - | + | + | - | - | 24 |
| - | - | + | + | + | + | + | - | + | - | 24 |
| - | - | + | - | - | - | - | - | + | - | 24 |
| - | + | + | + | + | - | + | + | - | - | 35 |
| + | + | + | - | - | - | + | - | - | + | 21 |
| + | + | + | + | + | + | + | + | + | + | 43 |
| + | + | + | + | + | + | - | + | + | + | 34 |
| + | + | + | + | + | + | + | + | + | + | 41 |
| - | + | + | - | + | + | - | + | - | + | 21 |
| - | - | + | - | - | - | - | - | - | - | 14 |
| - | + | + | - | + | - | - | - | - | - | 8 |
| - | - | + | - | - | - | - | - | - | - | 14 |
| - | - | + | - | - | + | - | - | - | + | 18 |
| - | + | + | + | - | - | - | + | - | - | 22 |
| + | + | + | + | + | + | + | + | + | - | 39 |
| - | - | + | + | + | + | + | + | + | - | 30 |
| + | + | + | + | + | + | + | + | + | - | 37 |
| + | - | + | - | + | - | - | - | + | - | 21 |
| + | + | - | + | - | - | - | - | + | + | 23 |
| - | + | + | - | + | + | - | - | + | + | 25 |
| - | - | + | + | + | - | + | + | + | - | 13 |
| - | + | + | - | + | - | - | + | - | - | 23 |
| - | + | + | - | + | - | - | + | + | + | 22 |
| + | + | + | + | + | + | + | + | + | + | 42 |
| 17 | 24 | 34 | 21 | 27 | 19 | 20 | 24 | 25 | 19 | 23.91 |
