# Supplementary material for: Finite element analysis of foot-insole biomechanics for insole design: a systematic review
Source: Front Bioeng Biotechnol. 2026 Oct 28;14:1994273. doi: 10.3389/fbioe.2026.1994273 (PMC13750745; doi:10.3389/fbioe.2026.1994273)
Supplement: Supplementary file 5 [file Supplementaryfile5.docx]

| **Appendix 5 Sensitivity of methodological quality classification** | | | | | | |
| --- | --- | --- | --- | --- | --- | --- |
| **Authors** | **Final score** | **Final classification** | **Assessor’s score (Gao)** | **Classification* (Gao)** | **Assessor’s score (Li)** | **Classification* (Li)** |
| Alsaleh et al., 2025 | 22 | Low quality | 21 | Low quality | 23 | High quality |
| Cracknell et al., 2025 | 28 | High quality | 28 | High quality | 27 | High quality |
| Lin et al., 2025 | 26 | High quality | 25 | High quality | 27 | High quality |
| Mala et al., 2025 | 15 | Low quality | 16 | Low quality | 13 | Low quality |
| Wang et al., 2025 | 27 | High quality | 27 | High quality | 28 | High quality |
| Zhang et al., 2025 | 19 | Low quality | 17 | Low quality | 16 | Low quality |
| Serrato-Pedrosa et al., 2024a | 27 | High quality | 26 | High quality | 24 | High quality |
| Serrato-Pedrosa et al., 2024b | 30 | High quality | 28 | High quality | 24 | High quality |
| Li et al., 2024 | 23 | High quality | 21 | Low quality | 23 | High quality |
| Du et al., 2024 | 22 | Low quality | 23 | High quality | 21 | Low quality |
| Geiger et al., 2023 | 32 | High quality | 29 | High quality | 27 | High quality |
| Nouman et al., 2023 | 21 | Low quality | 22 | Low quality | 21 | Low quality |
| Wang et al., 2023 | 22 | Low quality | 20 | Low quality | 22 | Low quality |
| Hu et al., 2023 | 18 | Low quality | 20 | Low quality | 19 | Low quality |
| Shaulian et al., 2022 | 22 | Low quality | 22 | Low quality | 22 | Low quality |
| AB Rashid et al., 2022 | 30 | High quality | 28 | High quality | 27 | High quality |
| Yang et al., 2022 | 35 | High quality | 33 | High quality | 31 | High quality |
| Peng et al., 2022 | 30 | High quality | 29 | High quality | 24 | High quality |
| Peng et al., 2021 | 34 | High quality | 31 | High quality | 31 | High quality |
| Jafarzadeh et al., 2021 | 22 | Low quality | 21 | Low quality | 23 | High quality |
| Zhu et al., 2021 | 17 | Low quality | 13 | Low quality | 13 | Low quality |
| Cao et al., 2021 | 23 | High quality | 22 | Low quality | 21 | Low quality |
| Nouman et al., 2021 | 23 | High quality | 24 | High quality | 22 | Low quality |
| Shaulian et al., 2021 | 24 | High quality | 26 | High quality | 20 | Low quality |
| Zhang et al., 2020b | 25 | High quality | 23 | High quality | 26 | High quality |
| Zhang et al., 2020a | 29 | High quality | 27 | High quality | 29 | High quality |
| Niu et al., 2020 | 17 | Low quality | 20 | Low quality | 15 | Low quality |
| Strayer et al., 2020 | 17 | Low quality | 19 | Low quality | 17 | Low quality |
| Tang et al., 2019 | 24 | High quality | 23 | High quality | 22 | Low quality |
| Zhang et al., 2018 | 24 | High quality | 21 | Low quality | 23 | High quality |
| Chanda et al., 2018 | 24 | High quality | 24 | High quality | 22 | Low quality |
| Zhang et al., 2017 | 24 | High quality | 23 | High quality | 23 | High quality |
| Zhou et al., 2017 | 21 | Low quality | 20 | Low quality | 21 | Low quality |
| Su et al., 2017 | 26 | High quality | 23 | High quality | 27 | High quality |
| Guo et al., 2016 | 23 | High quality | 23 | High quality | 21 | Low quality |
| Sarikhani et al., 2016 | 17 | Low quality | 16 | Low quality | 14 | Low quality |
| Chen et al., 2015 | 24 | High quality | 22 | Low quality | 23 | High quality |
| Liu et al., 2013 | 34 | High quality | 30 | High quality | 31 | High quality |
| Luo et al., 2011 | 21 | Low quality | 23 | High quality | 19 | Low quality |
| Cheung et al., 2008 | 27 | High quality | 28 | High quality | 22 | Low quality |
| Actis et al., 2008 | 19 | Low quality | 18 | Low quality | 16 | Low quality |
| Hsu et al., 2008 | 20 | Low quality | 20 | Low quality | 19 | Low quality |
| Goske et al., 2006 | 24 | High quality | 24 | High quality | 24 | High quality |
| Cheung et al., 2005 | 25 | High quality | 24 | High quality | 21 | Low quality |
| Chen et al., 2003 | 19 | Low quality | 19 | Low quality | 15 | Low quality |
| *High quality studies are studies with MQSSFE score ≥ 22.2; low quality studies are studies with MQSSFE score < 22.2. | | | | | | |
