# Supplementary material for: Finite element analysis of foot-insole biomechanics for insole design: a systematic review
Source: Front Bioeng Biotechnol. 2026 Oct 28;14:1994273. doi: 10.3389/fbioe.2026.1994273 (PMC13750745; doi:10.3389/fbioe.2026.1994273)
Supplement: Supplementary file 6 [file Supplementaryfile6.docx]

| **Appendix 6 Sensitivity of best-evidence synthesis to methodological quality classification** | | | | | |
| --- | --- | --- | --- | --- | --- |
| **Intervention** | **Outcome indicator** | **Main analysis*** | **Assessor (Gao)** | **Assessor (Li)** | **Conclusion stable** |
| Non-featured insole: an insole with a metatarsal pad, a toe pad, and arch support | Fascia stress | Limited | Limited | Limited | Yes |
|  | Bone stress | Limited | Limited | Limited | Yes |
| Non-featured insole | Plantar stress | Limited | Limited | Limited | Yes |
|  | Foot deformation | Limited | Limited | Limited | Yes |
|  | Metatarsal stress | Limited | Limited | Limited | Yes |
|  | Bone tuberosity stress | Limited | Limited | Limited | Yes |
| Non-featured insole: an insole with layered modular | Plantar pressure | Limited | Limited | Limited | Yes |
| Non-featured insole: an insole with pressurized bubbles | Plantar contact pressure | Limited | Limited | Limited | Yes |
| Non-featured insole: Yonex Active Pro Truactive, Victor VT-XD 8, Li-Ning L6200LA | Insole stress | Limited | Limited | Limited | Yes |
|  | Bone stress | Limited | Limited | Limited | Yes |
|  | Bone displacement | Limited | Limited | Limited | Yes |
|  | Bone rotation angle | Limited | Limited | Limited | Yes |
| Non-featured insole | Plantar pressure | Limited | Limited | Limited | Yes |
| Non-featured insole | Plantar pressure | Limited | Limited | Limited | Yes |
|  | Plantar frictional stress | Limited | Limited | Limited | Yes |
| Non-featured insole | Plantar pressure | Limited | Limited | Limited | Yes |
|  | Plantar frictional stress | Limited | Limited | Limited | Yes |
| Multi-hole-structured insole | Plantar pressure | Limited | Limited | Limited | Yes |
|  | Soft tissue stress | Limited | Limited | Limited | Yes |
| Single-hole-structured insole | Soft tissue stress (including the heel pad) | Moderate | Moderate | Moderate | Yes |
|  | Total stress concentration exposure of soft tissue | Limited | Limited | Limited | Yes |
|  | Plantar stress | Moderate | Moderate | Moderate | Yes |
|  | Heel pad strain | Limited | Limited | Limited | Yes |
|  | Strain energy density of heel pad | Limited | Limited | Limited | Yes |
| Multi-plug-structured insole | Plantar pressure | Limited | Limited | Limited | Yes |
| Single-plug-structured insole | Plantar pressure | Limited | Limited | Limited | Yes |
|  | Soft tissue stress | Limited | Limited | Limited | Yes |
|  | Total stress concentration exposure of soft tissue | Limited | Limited | Limited | Yes |
| Hole/plug-structured-insole+novel graded stiffness | Soft tissue stress | Limited | Limited | Limited | Yes |
|  | Total stress concentration exposure of soft tissue | Limited | Limited | Limited | Yes |
| Single-hole-structured insole+multi-parameter variations (insole material) | Plantar stress | Moderate | Moderate | Moderate | Yes |
| Variable stiffness insole | Plantar pressure | Moderate | Moderate | Moderate | Yes |
|  | Soft tissue stress | Limited | Limited | Limited | Yes |
|  | Bone stress | Limited | Limited | Limited | Yes |
|  | Cartilage stress | Limited | Limited | Limited | Yes |
|  | Total stress concentration exposure of soft tissue | Limited | Limited | Limited | Yes |
|  | Compressive stress  of heel pad | Limited | Limited | Limited | Yes |
|  | Tensile stress of skin | Limited | Limited | Limited | Yes |
| Variable stiffness insole+Conforming insole | Plantar pressure | Limited | Limited | Limited | Yes |
|  | Plantar contact area | Limited | Limited | Limited | Yes |
| Variable stiffness/thickness insole+Conforming insole | Plantar pressure | Limited | Limited | Limited | Yes |
| Variable stiffness/shape insole | Plantar pressure | Limited | Limited | Limited | Yes |
| Lateral wedge insole | Subtalar joint contact area | Limited | Limited | Limited | Yes |
|  | Subtalar joint stress | Limited | Limited | Limited | Yes |
|  | Plantar pressure | Limited | Limited | Limited | Yes |
|  | Joint contact pressure | Limited | Limited | Limited | Yes |
|  | Bone stress | Limited | Limited | Limited | Yes |
|  | Ligament stress | Limited | Limited | Limited | Yes |
|  | Cartilage stress | Limited | Limited | Limited | Yes |
|  | Meniscus stress | Limited | Limited | Limited | Yes |
|  | Meniscus contact force | Limited | Limited | Limited | Yes |
| Medial wedge insole | Plantar pressure | Limited | Limited | Limited | Yes |
|  | Bone stress | Limited | Limited | Limited | Yes |
|  | Ligament tension | Limited | Limited | Limited | Yes |
|  | Fascia stress | Limited | Limited | Limited | Yes |
| Arch support insole | Plantar pressure | Limited | Limited | Limited | Yes |
|  | Fascia strain | Limited | Limited | Limited | Yes |
|  | Ligament stress | Limited | Limited | Limited | Yes |
|  | Arch height | Limited | Limited | Limited | Yes |
|  | Cartilage stress | Limited | Limited | Limited | Yes |
|  | Plantar stress | Limited | Limited | Limited | Yes |
|  | Plantar strain | Limited | Limited | Limited | Yes |
|  | Plantar deformation | Limited | Limited | Limited | Yes |
| Arch support insole+multi-parameter variations | Arch height | Limited | Limited | Limited | Yes |
|  | Plantar pressure | Limited | Limited | Limited | Yes |
|  | Cartilage stress | Limited | Limited | Limited | Yes |
|  | Ligament stress | Limited | Limited | Limited | Yes |
|  | Plantar stress | Limited | Limited | Limited | Yes |
|  | Plantar strain | Limited | Limited | Limited | Yes |
|  | Plantar deformation | Limited | Limited | Limited | Yes |
| Conforming insole | Plantar stress | Limited | Limited | Limited | Yes |
|  | Plantar deformation | Limited | Limited | Limited | Yes |
|  | Insole stress | Limited | Limited | Limited | Yes |
|  | Metatarsal stress | Limited | Limited | Limited | Yes |
|  | Plantar pressure | Moderate | Moderate | Moderate | Yes |
|  | Fascia stress | Limited | Limited | Limited | Yes |
|  | Fascia strain | Limited | Limited | Limited | Yes |
| Conforming insole+multi-parameter variations (insole thickness and material hardness) | Plantar pressure | Limited | Limited | Limited | Yes |
|  | Plantar contact area | Limited | Limited | Limited | Yes |
|  | Bone stress (the forefoot and midfoot area) | Limited | Limited | Limited | Yes |
|  | Fascia strain | Limited | Limited | Limited | Yes |
|  | Metatarsal stress | Limited | Limited | Limited | Yes |
| Conforming insole+multi-parameter variations (insole thickness, material hardness, and heel’s height) | Plantar stress | Limited | Limited | Limited | Yes |
| Conforming insole+layered material design | Plantar stress | Limited | Limited | Limited | Yes |
| Arch support insole+wedge insole+multi-parameter variations (insole stiffness) | Plantar stress (surface) | Limited | Limited | Limited | Yes |
|  | Soft tissue stress | Limited | Limited | Limited | Yes |
|  | Cartilage stress | Limited | Limited | Limited | Yes |
|  | Fascia stress | Limited | Limited | Limited | Yes |
| Arch support insole+wedge insole+multi-parameter variations (insole stiffness) | Cartilage stress | Limited | Limited | Limited | Yes |
|  | Plantar pressure | Limited | Limited | Limited | Yes |
|  | Bone stress | Limited | Limited | Limited | Yes |
| Arch support insole+wedge insole+multi-parameter variations (insole stiffness and heel cup height) | Plantar pressure | Limited | Limited | Limited | Yes |
|  | Fascia strain | Limited | Limited | Limited | Yes |
| Partial insole (heel insole) | Plantar stress | Limited | Limited | Limited | Yes |
|  | Soft tissue stress | Limited | Limited | Limited | Yes |
|  | Fascia stress | Limited | Limited | Limited | Yes |
| Partial insole (metatarsal insole) | Plantar pressure | Limited | Limited | Limited | Yes |
|  | Soft tissue strain | Limited | Limited | Limited | Yes |
|  | Bone stress | Limited | Limited | Limited | Yes |
|  | Fascia tensile stress | Limited | Limited | Limited | Yes |
|  | Toe pad compressive strain | Limited | Limited | Limited | Yes |
| *Main analysis = final adjudicated methodological quality classification. | | | | | |
