# Supplementary material for: Finite element analysis of foot-insole biomechanics for insole design: a systematic review
Source: Front Bioeng Biotechnol. 2026 Oct 28;14:1994273. doi: 10.3389/fbioe.2026.1994273 (PMC13750745; doi:10.3389/fbioe.2026.1994273)
Supplement: Supplementary file 7 [file Supplementaryfile7.docx]

| **Appendix 7 Material properties on models** | | | | | | | |
| --- | --- | --- | --- | --- | --- | --- | --- |
| **Material** | | | **Authors** | **Elastic behavior** | **Young’s modulus (MPa)** | **Paisson’s ratio** | **Additional information** |
| **Models** | **Details** | |  |  |  |  |  |
| **Bone** | - | | Cracknell et al., 2025; Wang et al., 2025; Li et al., 2024; Du et al., 2024; Geiger et al., 2023; Nouman et al., 2023; Wang et al., 2023; Hu et al., 2023; Shaulian et al., 2022; AB Rashid et al., 2022; Jafarzadeh et al., 2021; Zhu et al., 2021; Cao et al., 2021; Nouman et al., 2021; Shaulian et al., 2021; Zhang et al., 2020a; Niu et al., 2020; Strayer et al., 2020; Zhou et al., 2017; Su et al., 2017; Guo et al., 2016; Sarikhani et al., 2016; Cheung et al., 2008; Hsu et al., 2008; Cheung et al., 2005 | Linearly elastic | 7300 | 0.3 | - |
|  |  |  | Zhang et al., 2020b; Zhang et al., 2017 | Linearly elastic | 7300 | 0.3 | Density (1500 kg·m^-3^) |
|  |  |  | Actis et al., 2008 | Linearly elastic | 7300 | - | - |
|  |  |  | Zhang et al., 2025; Yang et al., 2022; Chen et al., 2015 | - | 7300 | 0.3 | - |
|  |  |  | Tang et al., 2019 | Linearly elastic | 5500 | 0.3 | - |
|  |  |  | Chen et al., 2003 | Linearly elastic | 10000 | 0.34 | - |
|  |  |  | Peng et al., 2022; Peng et al., 2021 | - | 10000 | 0.34 | - |
|  | Cortical bone | | Lin et al., 2025 | Linearly elastic | 7300 | 0.3 | - |
|  | Cancellous bone | | Lin et al., 2025 | Linearly elastic | 100 | 0.3 | - |
| **Soft**  **tissue** | - | | Nouman et al., 2023;  Nouman et al., 2021 | Linearly elastic | 0.19 | 0.49 | - |
|  |  |  | Tang et al., 2019 | Linearly elastic | 0.45 | 0.49 |  |
|  |  |  | Su et al., 2017 | Linearly elastic | 1.19 | 0.48 | - |
|  |  |  | Wang et al., 2025; Lin et al., 2025; Du et al., 2024; Chen et al., 2003 | Linearly elastic | 1.15 | 0.49 | - |
|  |  |  | Zhang et al., 2017 | Linearly elastic | 1.15 | 0.49 | Density (937 kg·m^-3^) |
|  |  |  | Hsu et al., 2008; Cheung et al., 2005 | Linearly elastic | 0.15 | 0.45 | - |
|  |  |  | Zhou et al., 2017; Yang et al., 2022 | - | 0.15 | 0.45 | - |
|  |  |  | Li et al., 2024; Zhang et al., 2020b | Hyperelastic | Mooney-Rivlin | | - |
|  |  |  | Jafarzadeh et al., 2021; Geiger et al., 2023; Peng et al., 2022; Peng et al., 2021; Strayer et al., 2020; Cheung et al., 2008; Zhang et al., 2020a | Hyperelasti | 2^nd^-order Polynomial | | - |
|  |  |  | Sarikhani et al., 2016 | Hyperelastic | Ogden | | - |
|  |  |  | Cracknell et al., 2025; Chen et al., 2015 | Hyperelastic | 1^st^-order Ogden | | - |
|  |  |  | Liu et al., 2013 | Hyperelastic | Stress-strain data & Polynomial | | - |
|  |  |  | Shaulian et al., 2022;  Shaulian et al., 2021 | Visco-  hyperelastic | Prony & Noe-Hookean | | - |
|  | Healthy | | Zhu et al., 2021 | Linearly elastic | 0.45 | 0.49 | - |
|  |  |  | Wang et al., 2023; Hu et al., 2023; Niu et al., 2020; Cao et al., 2021 | Hyperelastic | 2^nd^-order Polynomial | | - |
|  | DM soft tissue | | Zhu et al., 2021 | Linearly elastic | 0.7 | 0.49 |  |
|  |  |  | Wang et al., 2023; Hu et al., 2023; Cao et al., 2021; Niu et al., 2020 | Hyperelastic | 2^nd^-order Polynomial | | - |
| **Plantar fascia** | - | | Li et al., 2024, Du et al., 2024; Jafarzadeh et al., 2021; Cheung et al., 2008; Hsu et al., 2008 | Linearly elastic | 350 | - | Cross sectional area (58.6 mm^2^) |
|  |  |  | Zhang et al., 2020a | - | 350 | - | Cross sectional area (58.6 mm^2^) |
|  |  |  | Zhang et al., 2020b; Zhang et al., 2017 | Linearly elastic | 350 | - | Cross sectional area (58.6 mm^2^), density (937 kg·m^-3^) |
|  |  |  | Guo et al., 2016 | Linearly elastic | 260-350 | - | Cross sectional area (290.7 mm^2^) |
|  |  |  | Cheung et al., 2005 | Linearly elastic | 350 | - | Cross sectional area (290.7 mm^2^) |
|  |  |  | Su et al., 2017 | Linearly elastic | 350 | 0.35 | Cross sectional area (290.7 mm^2^) |
|  |  |  | Yang et al., 2022 | - | 350 | - | Stiffness (39.1-650 N/mm) |
|  |  |  | Nouman et al., 2023;  Nouman et al., 2021 | Linearly elastic | 350 | 0.35 | - |
|  |  |  | Wang et al., 2023 | Linearly elastic | 350 | 0.45 | - |
|  |  |  | Peng et al., 2022; Peng et al., 2021 | - | 350 | 0.45 | - |
|  |  |  | Wang et al., 2025; Hu et al., 2023 | Linearly elastic | 350 | 0.4 | - |
|  |  |  | Chen et al., 2015 | - | 350 | 0.4 | - |
|  |  |  | Actis et al., 2008 | Linearly elastic | 85 | - | - |
|  |  |  | Niu et al., 2020 | Linearly elastic | - | - | Elastic coefficient (*k* = 1500 N/mm) |
| **Cartilage** | **-** | | Wang et al., 2023; Hu et al., 2023 | Linearly elastic | 50 | 0.1 | - |
|  |  |  | Shaulian et al., 2022; Chen et al., 2015 | Linearly elastic | 1.01 | 0.4 | - |
|  |  |  | Shaulian et al., 2021 | - | 1.01 | 0.4 | - |
|  |  |  | Zhang et al., 2025 | - | 1.01 | 0.45 | - |
|  |  |  | Yang et al., 2022 | - | 10 | 0.4 | - |
|  |  |  | Su et al., 2017; Guo et al., 2016; Chen et al., 2003 | Linearly elastic | 10 | 0.4 | - |
|  |  |  | Actis et al., 2008 | Linearly elastic | 10 | - | - |
|  |  |  | Zhang et al., 2020b | Linearly elastic | 1 | 0.4 | Density (1500 kg·m^-3^) |
|  |  |  | Wang et al., 2025; Du et al., 2024; Niu et al., 2020; Zhou et al., 2017; Hsu et al., 2008; Cheung et al., 2008; Cheung et al., 2005 | Linearly elastic | 1 | 0.4 | - |
|  |  |  | Lin et al., 2025 | Linearly elastic | 1 | 0.42 | - |
|  |  |  | Liu et al., 2013 | Linearly elastic | 12 | 0.45 | - |
|  | Arthritic cartilage | | Zhang et al., 2020a | - | 1 | 0.4 | - |
|  | Meniscus | | Zhang et al., 2017 | Linearly elastic | 59 | 0.49 | Density (2000 kg·m^-3^) |
|  |  |  | Liu et al., 2013 | Linearly elastic | 59 | 0.49 | - |
|  |  |  | Zhou et al., 2017 | - | 350 | - | - |
| **Ligament** | **-** | | Wang et al., 2023; Zhang et al., 2020a | - | 260 | - | Cross sectional area (18.4 mm^2^) |
|  | - | | Hu et al., 2023; Li et al., 2024; Du et al., 2024; Jafarzadeh et al., 2021; Cheung et al., 2008; Hsu et al., 2008; Cheung et al., 2005 | Linearly elastic | 260 | - | Cross sectional area (18.4 mm^2^) |
|  | - | | Wang et al., 2025; Chen et al., 2015 | Linearly elastic | 260 | 0.4 | Cross sectional area (18.4 mm^2^) |
|  | - | | Peng et al., 2022; Peng et al., 2021 | - | 260 | 0.4 | Cross sectional area (18.4 mm^2^) |
|  | - | | Zhang et al., 2020b; Zhang et al., 2017 | Linearly elastic | 260 | - | Cross sectional area (18.4 mm^2^), density (937 kg·m^-3^) |
|  | - | | Lin et al., 2025 | Linearly elastic | 260 | - | - |
|  | - | | Zhou et al., 2017 | - | 260 | - | - |
|  | Interosseous talocalcaneal, lateral talocalcaneal, etc. | | AB Rashid et al., 2022 | - | - | - | Stiffness (70-230 N/mm) |
|  | - | | Yang et al., 2022 | - | - | - | Stiffness (39.1-650 N/mm) |
|  | **-** | | Niu et al., 2020 | Linearly elastic | - | - | Elastic coefficient (*k* = 1500 N/mm) |
|  | **-** | | Chen et al., 2003 | Linearly elastic | 11.5 | - | Cross sectional area (3.16 mm^2^) |
|  | **-** | | Su et al., 2017 | - | 0-700 | 0.34 | Cross sectional area (18.4-260 mm^2^) |
|  | **-** | | Guo et al., 2016 | Linearly elastic | 0-700 | - | Cross sectional area (3.16-26.4 mm^2^) |
|  | **-** | | Liu et al., 2013 | Hyperelastic | Mooney-Rivlin | | - |
| **Skin** | - | | Serrato-Pedrosa et al., 2024a; Serrato-Pedrosa et al., 2024b | Linearly elastic | 0.2 | 0.485 | - |
|  |  |  | AB Rashid et al., 2022 | Linearly elastic | 1.15 | 0.49 | - |
|  |  |  | Su et al., 2017 | Linearly elastic | 1 | 0.4 | - |
|  |  |  | Shaulian et al., 2022;  Shaulian et al., 2021 | Visco-  hyperelastic | Prony & Neo-Hookean | | - |
|  |  |  | Zhang et al., 2025; Peng et al., 2022; Peng et al., 2021 | Hyperelastic | 1^st^-order Ogden | | - |
|  |  |  | Luo et al., 2011 | Hyperelastic | James-Green-Simpson | | - |
| **Muscle** | - | | Serrato-Pedrosa et al., 2024a;  Serrato-Pedrosa et al., 2024b | Linearly elastic | 0.06 | 0.495 | - |
|  |  |  | Serrato-Pedrosa et al., 2024a | Linearly elastic | 1.08 | 0.49 | - |
|  |  |  | Zhang et al., 2025 | - | 12 | 0.45 | - |
|  | Gastrocnemius | | Zhang et al., 2017 | Linearly elastic | 1.15 | 0.49 | Density (937 kg·m^-3^) |
| **Tendon** | Achilles Tendon | | Shaulian et al., 2022;  Shaulian et al., 2021 | Linearly elastic | 0.173 | 0.49 | - |
|  |  |  | Chen et al., 2015 | - | 816 | 0.3 | - |
|  | Flexor tendon | | Chen et al., 2015 | - | 450 | 0.3 | Cross sectional area (12.5 mm^2^) |
|  |  |  | Actis et al., 2008 | Linearly elastic | 15 | - | - |
| **Prosthesis** | Silicon rubber | | Guo et al., 2016 | Linearly elastic | 25 | 0.48 | - |
| **Foot** | - | | Alsaleh et al., 2025 | - | 50 | 0.4 | - |
|  | DM foot | | Mala et al., 2025; Chanda et al., 2018 | Linearly elastic | 483 | 0.4 | - |
| **Heel pad** | - | | Goske et al., 2006 | Hyperelastic | 1^st^-order Ogden | | - |
|  | - | | Zhang et al., 2025 | Hyperelastic | 1^st^-order Ogden | | - |
|  | - | | Luo et al., 2011 | Hyperelastic | 2^nd^-order Ogden | | - |
| **Ground** | - | | Alsaleh et al., 2025;  Serrato-Pedrosa et al., 2024b | Rigid | 210000 | 0.3 | - |
|  |  |  | Serrato-Pedrosa et al., 2024a; Su et al., 2017 | - | 210000 | 0.3 | - |
|  |  |  | Nouman et al., 2021 | - | 2100000 | 0.3 | - |
|  |  |  | Nouman et al., 2023 | Linearly elastic | 21000000 | 0.3 | - |
|  |  |  | Lin et al., 2025; Wang et al., 2025; Li et al., 2024; Wang et al., 2023; Hu et al., 2023; Cao et al., 2021; Jafarzadeh et al., 2021 | Linearly elastic | 17000 | 0.1 | - |
|  |  |  | Yang et al., 2022 | - | 17000 | 0.1 | - |
|  |  |  | Zhang et al., 2020b | - | 17000 | 0.1 | Density (5000 kg·m^-3^) |
|  |  |  | Zhang et al., 2017 | Linearly elastic | 17000 | 0.1 | Density (5000 kg·m^-3^) |
|  |  |  | Zhang et al., 2020a; Zhang et al., 2018 | Rigid | 17000 | 0.1 | - |
|  |  |  | Niu et al., 2020 | Rigid | 1000000 | 0.1 | - |
|  |  |  | Hsu et al., 2008 | Linearly elastic | 1000000 | 0.1 | - |
|  |  |  | Zhang et al., 2025 | Rigid | 205000 | 0.49 | - |
|  |  |  | Zhu et al., 2021 | - | 9.4 | 0.1 |  |
|  | Upper layer | | Du et al., 2024 | - | 17000 | 0.1 | - |
|  |  |  | Cheung et al., 2008 | - | 17000 | - | - |
|  | Lower layer | | Cheung et al., 2008 | - | 10000000 |  | - |
| **Test insole** | - | - | Zhang et al., 2020b; Zhang et al., 2018 | - | 1 | - | - |
|  | - | - | Zhu et al., 2021 | - | 1000 | - | - |
|  | - | - | Li et al., 2024 | Linearly elastic | 11.7 | 0.45 | - |
|  | EVA | (Shore A-3) | Wang et al., 2023 | Hyperelastic | 2^nd^-order Ogden | | - |
| **Insole** | - | - | Li et al., 2024 | Linearly elastic | 1.04-74 | 0.45 | - |
|  | - | - | Wang et al., 2025 | Linearly elastic | 84 | 0.45 | - |
|  | - | - | Sarikhani et al., 2016 | Linearly elastic | 0.3, 1000, 2000 | 0.4 | - |
|  | - | - | Lin et al., 2025 | - | 100, 260, 1000, 3000, 5000, 500000 | 0.45 | - |
|  | - | - | Peng et al., 2022 | - | 3, 5, 7 | - | - |
|  | - | - | Zhang et al., 2020b; Zhang et al., 2020a | - | 1.0, 1.5, 2.5 | - | - |
|  | - | - | Zhou et al., 2017 | - | 1000 | 0.4 | - |
|  | - | - | Chanda et al., 2018 | Hyperplastic | Veronda-Westmann | | - |
|  | - | - | Luo et al., 2011 | Hyperelastic | 2^nd^-order Ogden | | - |
|  | - | Amfit | Nouman et al., 2021 | - | 8.97 | 0.39 | - |
|  | - | Yonex Active Pro Truactive, etc. | AB Rashid et al., 2022 | Linearly elastic | 50 | 0.4 | - |
|  | Firm | - | Nouman et al., 2023 | Linearly elastic | 1000 | 0.4 | - |
|  | Fluid | - | Cracknell et al., 2025 | - | Permeability constitutive material model | | - |
|  | EVA graded material | - | Shaulian et al., 2022 | Hyperelastic | Neo-Hookean | | - |
|  | EVA insole cover | - | Shaulian et al., 2022 | Hyperelastic | Neo-Hookean | | - |
|  | initial & EVA graded material | - | Jafarzadeh et al., 2021 | Linearly elastic | 0.4-74 | 0.3 | - |
|  | PU & EVA | - | Zhang et al., 2017 | Linearly elastic | 0.5, 1.0, 1.5, 2.0 | - | Density (2000 kg·m^-3^) |
|  | Rubber | - | Alsaleh et al., 2025 | Linearly elastic | 6 | 0.45 | - |
|  | PVC | - | Strayer et al., 2020 | Linearly elastic | 3, 5, 10 | 0.4 | - |
|  | PP | - | Cheung et al., 2005 | - | 1000 | 0.4 | - |
|  | PPT | - | Chen et al., 2003 | Hyperelastic | 1^st^-order Ogden | | - |
|  | TPC | - | Geiger et al., 2023 | Linearly elastic | 4 | 0.48 | - |
|  | TPI & EVA | SM-1R | Hsu et al., 2008 | Linearly elastic | 0.4 | 0.2 | - |
|  | Ther-  mocork | - | Chen et al., 2003 | Hyperelastic | 1^st^-order Ogden | | - |
|  | Latex | - | Yang et al., 2022 | Hyperelastic | 1^st^-order Ogden | | - |
|  | PU | Poron Cushioning | Goske et al., 2006 | Hyperelastic | 1^st^-order Ogden | | - |
|  |  | Poron_L 24 (Shore A-10) | Cao et al., 2021 | Hyperelastic | 2^nd^-order Ogden | | - |
|  |  | Poron_L32 (Shore A-20) | Guo et al., 2016 | Hyperelastic | 2^nd^-order Ogden | | - |
|  |  | Poron_L 24 (Shore A-10) | Cheung et al., 2008 | Hyperelastic | 2^nd^-order Ogden | | - |
|  |  | Poron_L32 (Shore A-20) | Cheung et al., 2008 | Hyperelastic | 2^nd^-order Ogden | | - |
|  |  | - | Cheung et al., 2005 | - | 0.3 | 0.4 | - |
|  |  | - | Mala et al., 2025 | - | 0.04899 | 0.2793 | Density (31.94 kg·m^-3^) |
|  | PE | Plastazote | Chen et al., 2015 | Hyperelastic | 2^nd^-order Ogden | | - |
|  |  | Plastazote | Nouman et al., 2023 | Linearly elastic | 0.45 | 0.38 | - |
|  |  | Plastazote medium density | Chen et al., 2003 | Hyperelastic | 1^st^-order Ogden | | - |
|  | EVA | - | Du et al., 2024 | - | 84 | 0.4 | - |
|  |  | (Shore A-65) | Mala et al., 2025 | - | 7.593 | 0.4799 | Density (950 kg·m^-3^) |
|  |  | (Shore A-85) | Mala et al., 2025 | - | 24.49 | 0.4799 | Density (950 kg·m^-3^) |
|  |  | (Shore A-95/D-50) | Mala et al., 2025 | - | 79.37 | 0.4799 | Density (935 kg·m^-3^) |
|  |  | - | Alsaleh et al., 2025 | Linearly elastic | 1.66 | 0.49 |  |
|  |  | - | Nouman et al., 2023 | Linearly elastic | 5 | 0.4 | - |
|  |  | - | Nouman et al., 2021 | - | 5 | 0.4 | - |
|  |  | Nora Lunalastike | Nouman et al., 2023 | Linearly elastic | 1.04 | 0.25 | - |
|  |  | High density | Cheung et al., 2005 | - | 1 | 0.4 |  |
|  |  | - | Zhang et al., 2018 | - | 2 | 0.35 | - |
|  |  | - | Shaulian et al., 2022,  Shaulian et al., 2021 | Hyperelastic | Neo-Hookean | | - |
|  |  | - | Yang et al., 2022 | Hyperelastic | Mooney-Rivlin | | - |
|  |  | Microcel Puff | Goske et al., 2006; Chen et al., 2003 | Hyperelastic | 1^st^-order Ogden | | - |
|  |  | Microcel Puff Lite | Goske et al., 2006 | Hyperelastic | 1^st^-order Ogden | | - |
|  |  | Nora SLW (Shore A-30) | Cheung et al., 2008 | Hyperelastic | 2^nd^-order Ogden | | - |
|  |  | Nora SL (Shore A-40) | Cheung et al., 2008 | Hyperelastic | 2^nd^-order Ogden | | - |
|  | TPU | - | Alsaleh et al., 2025; Nouman et al., 2023 | Linearly elastic | 11 | 0.45 | - |
|  |  | - | Nouman et al., 2021; Serrato-Pedrosa et al., 2024a; Serrato-Pedrosa et al., 2024b | - | 11 | 0.45 | - |
|  |  | - | Hu et al., 2023 | Linearly elastic | 11.7 | 0.45 | - |
|  |  | - | Wang et al., 2023; Zhu et al., 2021 | - | 11.7 | 0.45 | - |
|  |  | PolyFlex_TDS-v1 | Tang et al., 2019 | - | 11.7 | 0.45 | - |
|  |  | IROPRINT F80213 | Peng et al., 2021 | - | 7.8 | 0.35 | - |
| Midsole | - | - | AB Rashid et al., 2022 | - | 2.49 | 0.35 | - |
|  | - | - | Peng et al., 2022; Peng et al., 2021 | - | 5 | 0.4 | - |
|  | EVA | (Asker C-45, 60, 70) | Yang et al., 2022 | Hyperelastic | Mooney-Rivlin | | - |
|  |  | Microcell puff | Chen et al., 2015 | Hyperelastic | 2^nd^-order Ogden | | - |
|  |  | Nora SLW (Shore A-30) | Cheung et al., 2008 | Hyperelastic | 2^nd^-order Ogden | | - |
|  |  | Nora SL (Shore A-40) | Cheung et al., 2008 | Hyperelastic | 2^nd^-order Ogden | | - |
|  |  | Nora AL (Shore A-50) | Cheung et al., 2008 | Hyperelastic | 2^nd^-order Ogden | | - |
|  | PU | Poron_L32 (Shore A-20) | Cheung et al., 2008 | Hyperelastic | 2^nd^-order Ogden | | - |
|  | Crepe | - | Goske et al., 2006 | Hyperelastic | 1^st^-order Ogden | | - |
| **Outsole** | - | - | AB Rashid et al., 2022 | - | 25 | 0.42 | - |
|  | Rubber | - | Yang et al., 2022 | - | 8 | 0.47 | - |
| **Sole** | - | - | Geiger et al., 2023 | Linearly elastic | 1000 | 0.42 | - |
|  | EVA | Nora AL  (Shore A-50) | Cheung et al., 2008 | Hyperelastic | 2^nd^-order Ogden | | - |
| **Upper** | - | - | Yang et al., 2022 | - | 11.76 | 0.35 | - |
|  | Leather | - | Goske et al., 2006 | Linearly elastic | 200000 | 0.3 | - |
| **Heel cup** | EVA | - | Yang et al., 2022 | Hyperelastic | Mooney-Rivlin | | - |
|  | Latex | - | Yang et al., 2022 | Hyperelastic | 1^st^-order Ogden | | - |
| **Plug** | - | - | Shaulian et al., 2022 | Hyperelastic | Neo-Hookean | | - |
|  | - | (Shore A-9, 20, 29) | Geiger et al., 2023 | - | 0.5, 1.0, 1.5 | - | - |
| **DM, Diabetes Mellitus; PPT, Polypropylene Terephthalate; TPU, Thermoplastic Polyurethane; EVA, Ethylene Vinyl Acetate; TPC, Thermoplastic Copolyester; PE, Polyethylene; PVC, Polyvinyl Chloride; PU, Polyurethane; TPI, Trans-polyisoprene; PP, Polypropylene;** | | | | | | | |
